# Nrf2 activation reprograms macrophage intermediary metabolism and suppresses the type I interferon response

**DOI:** 10.1016/j.isci.2022.103827

**Published:** 2022-01-30

**Authors:** Dylan G. Ryan, Elena V. Knatko, Alva M. Casey, Jens L. Hukelmann, Sharadha Dayalan Naidu, Alejandro J. Brenes, Thanapon Ekkunagul, Christa Baker, Maureen Higgins, Laura Tronci, Efterpi Nikitopolou, Tadashi Honda, Richard C. Hartley, Luke A.J. O’Neill, Christian Frezza, Angus I. Lamond, Andrey Y. Abramov, J. Simon C. Arthur, Doreen A. Cantrell, Michael P. Murphy, Albena T. Dinkova-Kostova

**Affiliations:** 1 School of Biochemistry and Immunology, Trinity Biomedical Sciences Institute, Trinity College Dublin, Dublin, Ireland; 2 Medical Research Council Cancer Unit, University of Cambridge, Cambridge, UK; 3 Division of Cellular Medicine, School of Medicine, University of Dundee, Ninewells Hospital and Medical School, James Arrott Drive, Dundee, Scotland, UK; 4 Medical Research Council Mitochondrial Biology Unit, University of Cambridge, Cambridge, UK; 5 Division of Cell Signalling and Immunology, School of Life Sciences, University of Dundee, Dundee, Scotland, UK; 6 Centre for Gene Regulation and Expression, School of Life Sciences, University of Dundee, Dundee, Scotland, UK; 7 Department of Chemistry and Institute of Chemical Biology & Drug Discovery, Stony Brook University, Stony Brook, NY, USA; 8 School of Chemistry, University of Glasgow, Glasgow, UK; 9 Department of Clinical and Movement Neurosciences, University College London Queen Square Institute of Neurology, London, UK; 10 Department of Pharmacology and Molecular Sciences and Department of Medicine, Johns Hopkins University School of Medicine, Baltimore, MD, USA

**Keywords:** Biochemistry, Immunology, Proteomics

## Abstract

To overcome oxidative, inflammatory, and metabolic stress, cells have evolved cytoprotective protein networks controlled by nuclear factor-erythroid 2 p45-related factor 2 (Nrf2) and its negative regulator, Kelch-like ECH associated protein 1 (Keap1). Here, using high-resolution mass spectrometry we characterize the proteomes of macrophages with altered Nrf2 status revealing significant differences among the genotypes in metabolism and redox homeostasis, which were validated with respirometry and metabolomics. Nrf2 affected the proteome following lipopolysaccharide (LPS) stimulation, with alterations in redox, carbohydrate and lipid metabolism, and innate immunity. Notably, Nrf2 activation promoted mitochondrial fusion. The Keap1 inhibitor, 4-octyl itaconate remodeled the inflammatory macrophage proteome, increasing redox and suppressing type I interferon (IFN) response. Similarly, pharmacologic or genetic Nrf2 activation inhibited the transcription of IFN-β and its downstream effector IFIT2 during LPS stimulation. These data suggest that Nrf2 activation facilitates metabolic reprogramming and mitochondrial adaptation, and finetunes the innate immune response in macrophages.

## Highlights

• High-resolution proteome and metabolome of macrophages with altered Nrf2 status
• Nrf2 regulates macrophage intermediary metabolism and mitochondrial adaptation
• Genetic Nrf2 activation with Keap1-KD suppresses IFN-β and the type I IFN response
• Nrf2 activation with electrophilic Keap1 modifiers suppresses the type I IFN response

## Graphical abstract

**Figure:**
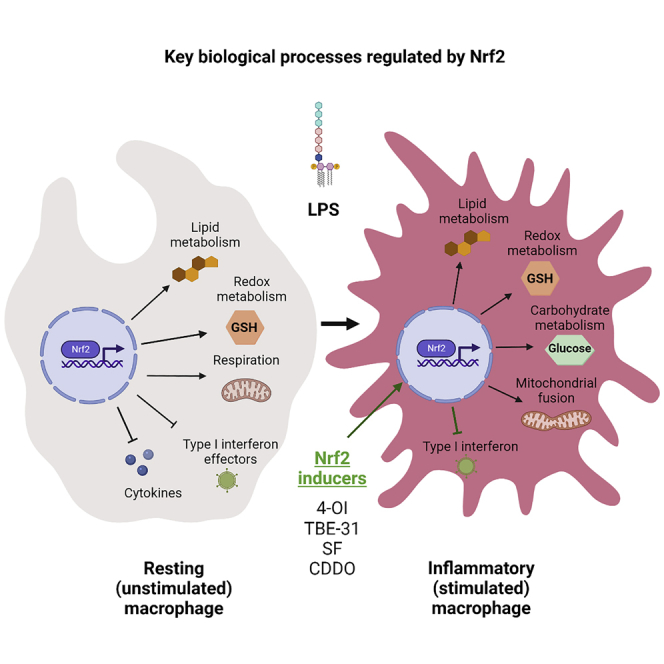

## Introduction

The transcription factor (TF) nuclear factor-erythroid 2 p45-related factor 2 (Nrf2, gene name *Nfe2l2*), and its negative regulator Kelch-like ECH associated protein 1 (Keap1) are at the interface of redox and intermediate metabolism (Hayes and Dinkova-Kostova, 2014; Yamamoto et al., 2018), and have a complex, but incompletely understood, function in infection, inflammation, and immunity (Cuadrado et al., 2020). This is not surprising considering that infection and inflammation cause disturbances in cellular redox homeostasis, which is restored by the upregulation of Nrf2-target proteins (Hayes and Dinkova-Kostova, 2014). 4-Octyl itaconate (4-OI), a derivative of the immunometabolite itaconate that activates Nrf2 via Keap1 alkylation, suppresses certain pro-inflammatory cytokines in macrophages *in vitro* and is protective in an LPS lethality model *in vivo* (Mills et al., 2018). Furthermore, genetic and pharmacologic Nrf2 activation is considered anti-inflammatory and facilitates the resolution of inflammation (Dayalan Naidu et al., 2018; Kobayashi et al., 2016).

Nrf2 also activates the transcription of genes important for macrophage function, such as macrophage receptor with collagenous structure (MARCO) (Harvey et al., 2011), a receptor required for bacterial phagocytosis, cluster of differentiation 36 (CD36) (Maruyama et al., 2008), a scavenger receptor for oxidized low-density lipoproteins, and the virus surveillance mediator interleukin-17D (IL-17D) (Saddawi-Konefka et al., 2016). In cancer cells, Nrf2 promotes the replication of the vesicular stomatitis virus Δ51, facilitating oncolytic infection (Olagnier et al., 2017). By contrast, Nrf2 is inactivated by herpes simplex virus 1 (HSV-1) or severe acute respiratory syndrome coronavirus 2 (SARS-CoV-2), while the Nrf2 activators 4-octyl itaconate (4-OI), sulforaphane, 2-cyano-3,10-dioxooleana-1,9(11)-dien-28-oic acid (CDDO) (Figure S1), and its C-28 methyl ester (CDDO-Me, bardoxolone methyl) inhibit the replication of these viruses, correlating with increased resistance to infection (Olagnier et al., 2020; Ordonez et al., 2021; Sun et al., 2021; Wyler et al., 2019).

Although the downregulation of pro-inflammatory responses by Nrf2 activation is consistently observed in cells and in animal and human tissues (Dayalan Naidu et al., 2018; Knatko et al., 2015; Kobayashi et al., 2016; Liu et al., 2020; Thimmulappa et al., 2006), global in-depth insights of the effect of Nrf2 on macrophages and their responses to inflammatory stimuli are lacking. In this study, we asked how Nrf2 affects the proteome and metabolome of differentiated macrophages, comparing both resting and stimulated states. We identified a central role for Nrf2 in regulating the metabolic landscape of macrophages, influencing a plethora of processes involved in redox, carbohydrate, and lipid metabolism. Moreover, we found a role for Nrf2 in regulating respiration and mitochondrial fusion in activated macrophages. Additionally, Nrf2 was found to have a key regulatory role on the innate immune response, suppressing interferon-beta (IFN-β). Finally, we identified a crucial role for Nrf2 in mediating the suppressive effects of electrophilic Keap1 modifiers (Figure S1) on IFN-β and IFN effector proteins. These findings place Nrf2 at the center of macrophage metabolism and have major implications for our understanding of Nrf2 biology and how it regulates the innate immune response.

## Results

### The proteomes of resting and activated macrophages with altered Nrf2 status

**Figure 1:**
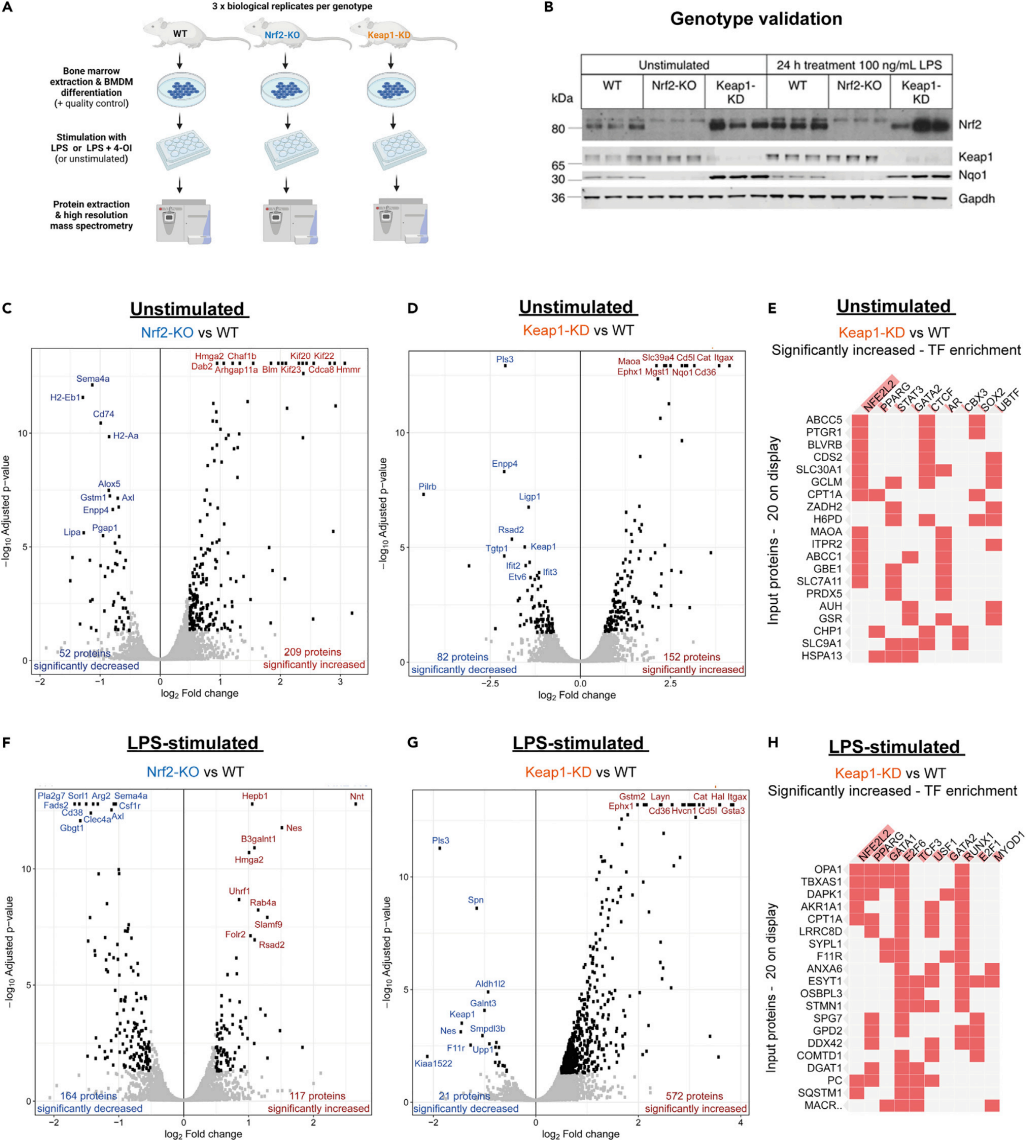
Nrf2 remodels the proteome in resting and activated macrophages (A) Experimental design and workflow. (B) Validation of WT, Nrf2 and Keap1-KO genotypes (n = 3 biological replicates). (C) Volcano plot of unstimulated Nrf2-KO compared to WT. (D) Volcano plot of unstimulated Keap1-KD compared to WT. (E) Transcription factor (TF) enrichment of increased targets in unstimulated Keap1-KD compared to WT. (F) Volcano plot of LPS-stimulated Nrf2-KO compared to WT. (G) Volcano plot of LPS-stimulated Keap1-KD compared to WT. (H) TF enrichment of increased targets in LPS-stimulated Keap1-KD compared to WT. (E, H) ORA by Enrichr. Top transcription factors ranked according to combined enrichment score (p value and *Z* score) using ENCODE and ChEA databases. (C-D, F-G) (n = 3 biological replicates). Cut-offs - log_2_FC = 0.5; FDR <0.05, determined using t statistics. (E, F) (combined score – p value and *Z* score)

To understand how Nrf2 regulates macrophage biology, we used high-resolution mass spectrometry (MS) to characterize the proteomes of bone marrow-derived macrophages (BMDMs) differentiated *ex vivo* from bone marrow cells isolated from, respectively, wild-type (WT), Keap1-knockdown (Keap1-KD, expressing ∼70% lower levels of Keap1 compared to WT, and consequently high Nrf2 levels) and Nrf2-knockout (Nrf2-KO, expressing transcriptionally inactive Nrf2-β-galactosidase fusion protein) mice (Knatko et al., 2020) both in a resting (unstimulated) state and following LPS stimulation (100 ng/mL, 24 h) (Figure 1A). We confirmed the genotype of the macrophages by immunoblotting for Nrf2, Keap1, and the prototypical Nrf2 target, NAD(P)H:quinone oxidoreductase 1 (Nqo1) (Figure 1B). Interestingly, we observed some small but significant changes in cell size among the genotypes, whereby Nrf2-KO and Keap1-KD resulted in a decrease and an increase, respectively (Figure S2A).

The levels of Nrf2 increased following macrophage activation (Figure 1B), consistent with previous findings (Mills et al., 2018). Differential expression analysis in the Nrf2-KO and Keap1-KD genotypes revealed substantial changes to the proteome in both resting and activated macrophages (Figures 1C, 1D, 1F-1G, Tables S1 and S2). Nrf2 targets were enriched in the significantly increased proteins of Keap1-KD macrophages (Figures 1E, 1H, and S2B). Similarly, TF enrichment analysis of proteins significantly decreased with Nrf2-KO confirmed enrichment for Nrf2 targets (Figure S2C). Of note, Nrf2 disruption led to a greater decrease in the number of proteins in LPS-stimulated macrophages (Figure 1F), whereas Keap1-KD led to a substantial increase (Figure 1G), which suggests that Nrf2 stabilization is an important feature of the LPS response. Enrichment for innate immune pathways, including cytokine and IFN signaling, was detected in LPS-activated WT macrophages (Figure S2D, Table S3). Although certain cytokines and IFNs, such as interleukin-1β (IL1β), IL-6, TNF, and IFN-β, were not detected (presumably due to secretion and/or technical detection limitations), LPS did increase the transcript levels of *Il1b* (Figure S2E), validating the ability of LPS to promote macrophage activation. Furthermore, we confirmed the ability of 4-OI to inhibit *Il1b* in part via Nrf2 (Figure S2E). Finally, Keap1-KD led to a suppression of *Il1b*, showing that the genetic activation of Nrf2 also suppresses specific pro-inflammatory cytokines.

### Nrf2 is a critical regulator of metabolism and innate immune pathways in resting macrophages

**Figure 2:**
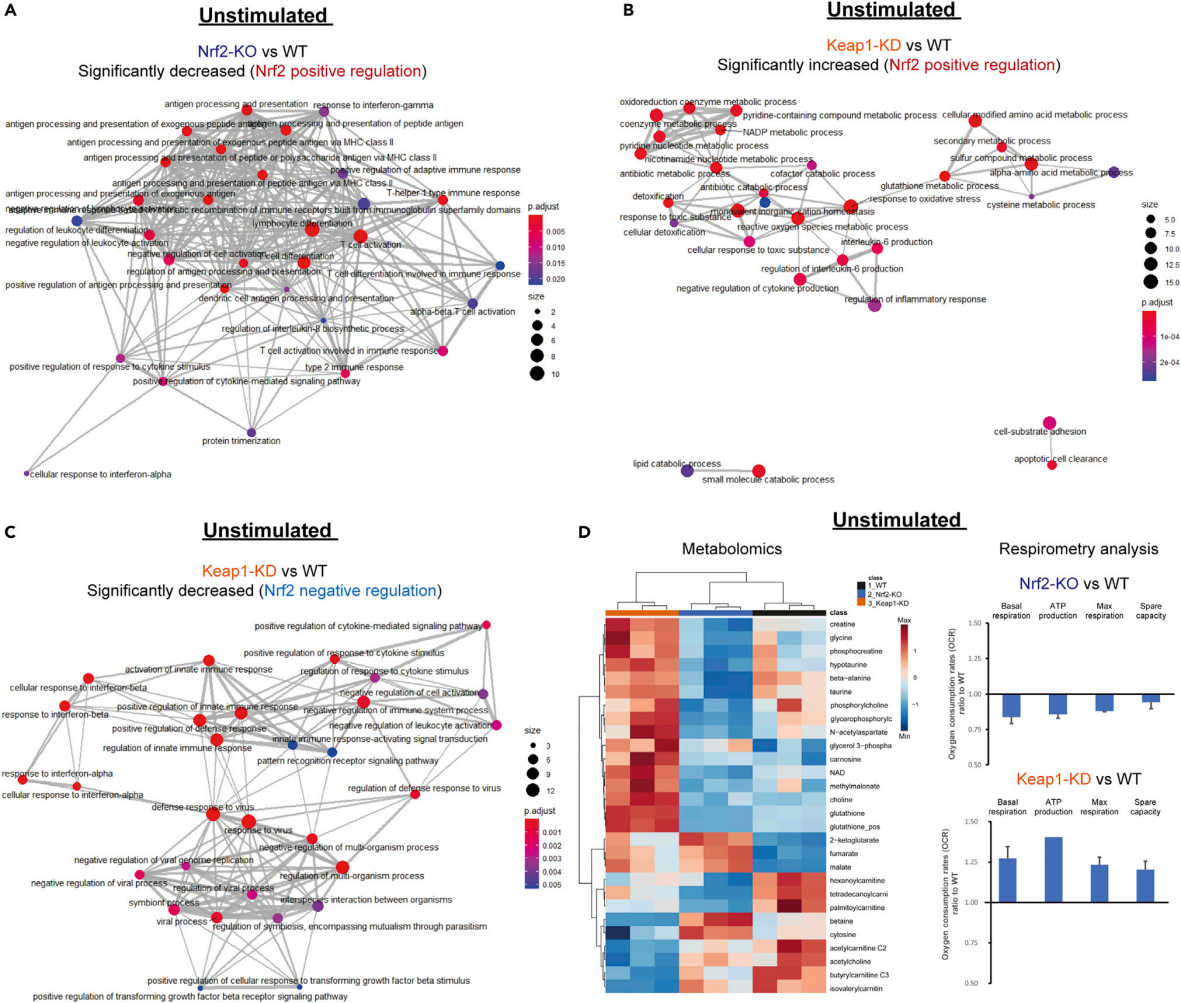
Nrf2 suppresses proteins involved in anti-viral immunity and cytokine production, while maintaining cellular redox metabolism (A) Enrichment map of GO: biological processes of Nrf2 positively regulated targets (Nrf2-KO vs WT). (B) Enrichment map of GO: biological processes of Nrf2 positively regulated targets (Keap1-KD vs WT). (C) Enrichment map of GO: biological processes of Nrf2 negatively regulated targets – Keap1-KD versus WT (A-C) ORA by clusterProfiler, FDR correction by Bonferroni test. (D) Heatmap of significantly altered metabolites (n = 3 biological replicates) and oxygen consumption rates (OCR) (representative of three biological replicates). p value determined by one-way ANOVA corrected for multiple comparisons by Tukey statistical test. Cut off – FDR <0.05.

To better understand the biological processes that Nrf2 regulates in the resting state, we performed an over-representation analysis (ORA) on the significantly decreased (Nrf2 positive regulation) (Figures 2A and S3A) and increased (Nrf2 negative regulation) (Figure S3D) proteins of Nrf2-KO macrophages. The analysis of positively regulated processes revealed a large functional cluster, which included antigen processing and presentation and factors involved in regulating IFN and T cell responses (Figure 2A). Hepoxilin metabolic enzymes that synthesize inflammatory polyunsaturated fatty acids (PUFAs) were also significantly increased (Figure S3A). Lipid oxidation and glutathione (GSH)-related enzymes, as well as positive regulators of type 2 immune response, were also increased, although no significant enrichment of these processes in the resting state was observed (Figure S3A). Amongst the targets negatively regulated by Nrf2, two functional clusters were observed, which included proteins involved in cell division and DNA replication and repair (Figure S3D).

In Keap1-KD macrophages, ORA of the significantly increased proteins (Nrf2 positive regulation) identified three functional clusters (Figure 2B). The major cluster included biological processes involved in oxidative stress, sulfur metabolism, and inflammatory response regulators, while the final two clusters identified were lipid catabolism and cell adhesion (Figure 2B). The most significantly enriched processes included GSH transport, GSH metabolism, transulfuration pathway, and the response to low-density lipoprotein (Figure S3B). In contrast, ORA of negatively regulated processes revealed two interconnected functional clusters, including proteins involved in anti-viral immune response and positive regulators of the innate immune response (Figure 2C). Interestingly, type I IFN effectors were among the most significantly enriched downregulated targets with Nrf2 activation (Figure S3C). Thus, our proteomic analysis suggests that Nrf2 is required to maintain cellular redox and lipid homeostasis, while modulating distinct innate immune effectors, notably decreasing type I IFN signaling.

Changes in the resting macrophage proteome conferred by either Nrf2 disruption or Keap1 knockdown were confirmed using MS-based label-free data-independent acquisition (DIA) proteomics (Figures S4A–S4E). In agreement with the increase in cell size with Keap1 disruption (Figure S2A), total protein content, as estimated using the proteomic ruler (Wisniewski et al., 2014), was increased in Keap1-KD macrophages (Figure S4A). TMT and DIA demonstrated significant overlap in protein hits identified, although TMT was more sensitive (Figure S4B). Significantly differentially regulated targets in Keap1-KD and Nrf2-KO relative to WT showed good agreement between TMT and DIA datasets (Figures S4C and S4D). However, DIA better detected a decrease in prototypical redox-regulated enzymes in Nrf2-KO macrophages at resting state (Figures S4C and S4D). In agreement with the TMT proteomics (Figures 1E and 1H), ORA analysis of differentially regulated targets in Keap1-KD from the DIA dataset demonstrated significant enrichment for the TF Nrf2 (Figure S4E).

**Figure 3:**
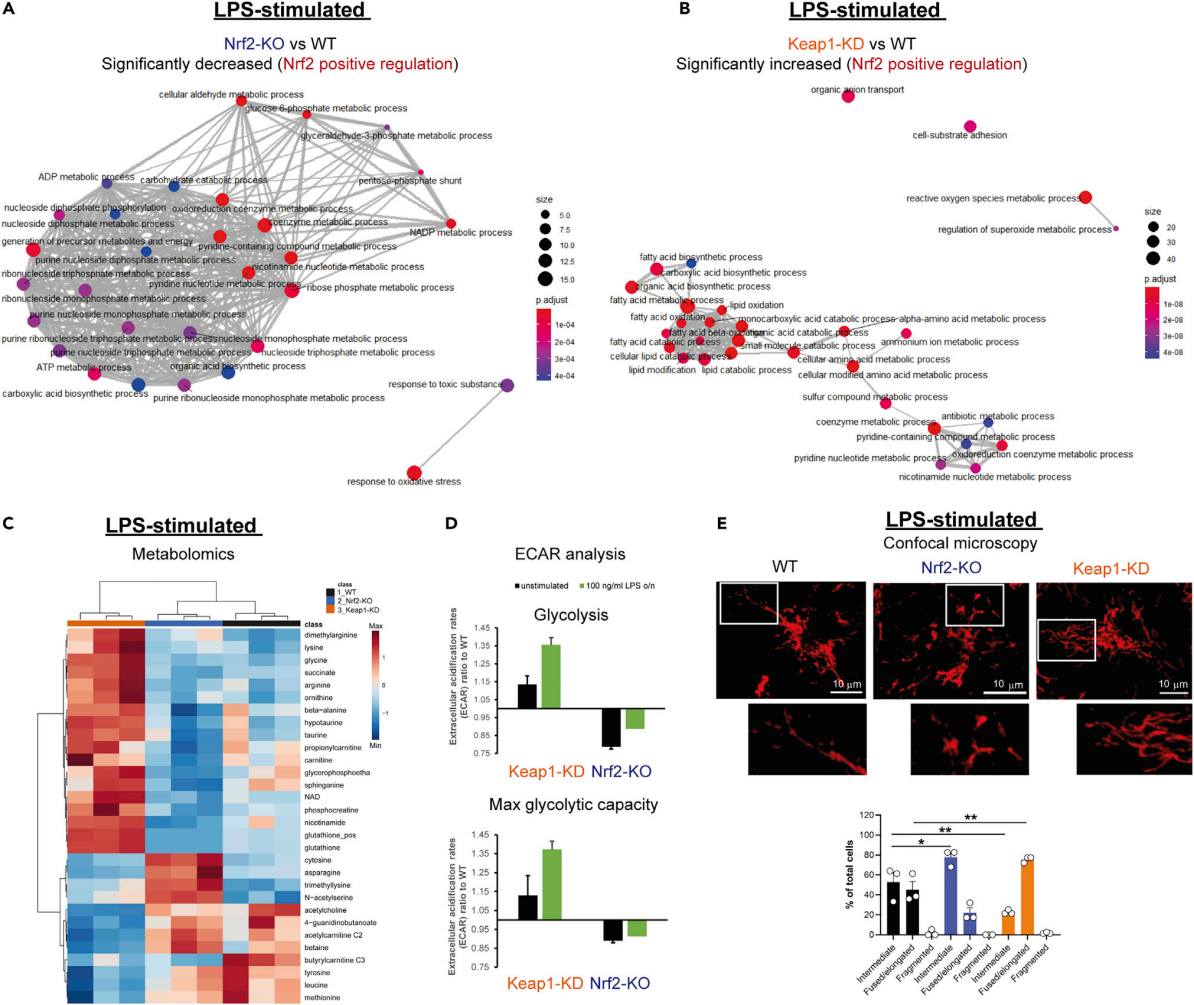
Nrf2 is a central regulator of metabolism, mitochondrial adaptation, and immune effector functions in inflammatory macrophages (A) Enrichment map of GO: biological processes of Nrf2 positively regulated targets (Nrf2-KO vs WT). (B) Enrichment map of GO: biological processes of Nrf2 positively regulated targets (Keap1-KD vs WT) (A-B) ORA by clusterProfiler, FDR correction by Bonferroni test. (C) Heatmap of significantly altered metabolites (n = 3 biological replicates). p value determined by one-way ANOVA corrected for multiple comparisons by Tukey statistical test. Cut off – FDR <0.05. (D) Extracellular acidification rates (ECAR) (representative of three biological replicates). (E) Confocal microscopy of mitochondrial morphology using TOM20 (images are representative, bar plot n = 3 biological replicates). Scale bar 10 μm. Data are mean ± SEM p value determined by one-way ANOVA, corrected for multiple comparisons by Tukey statistical test. p < 0.05∗; p <0.01∗∗; p <0.001∗∗∗.

To validate a role for Nrf2 in regulating metabolism, we performed liquid chromatography-mass spectrometry (LC-MS)-based metabolomic analysis of resting WT, Nrf2-KO, and Keap1-KD macrophages (Figure 2D, left panel; Table S8). A clear segregation of the metabolome according to genotype was observed (Figures 2D, 3C, and S3E), validating a role for Nrf2 in regulating basal macrophage metabolism. Notably, Nrf2 activation significantly increased antioxidant metabolites, including GSH, hypotaurine, taurine, β-alanine, and carnosine, whereas Nrf2 disruption significantly decreased intracellular GSH, taurine, hypotaurine and β-alanine (Figure 2D). Indeed, the two subunits (Gclc and Gclm) of the rate-limiting GSH biosynthetic enzyme glutamate cysteine ligase, as well as Nqo1 and glutathione reductase (Gsr), were decreased with Nrf2-KO and increased with Keap1-KD (Figure S3F). Furthermore, both Nrf2 activation and disruption led to significant alterations in mitochondrial metabolites, such as those involved in fatty acid oxidation (FAO) (carnitine, palmitoylcarnitine, hexanoylcarnitine, and tetradecanoylcarnitine), the TCA cycle (fumarate, malate, and 2-ketoglutarate), and bioenergetics (NAD, creatine, phosphocreatine). These findings suggest an involvement of Nrf2 in regulating mitochondrial metabolism in macrophages.

To confirm these observations using MS-independent methods, we performed a respirometry analysis of oxygen consumption rates (OCR) in all three genotypes. This identified a role for Nrf2 in regulating mitochondrial respiration (Figure 2D, right panel). Nrf2 activation increased the basal respiration rates associated with ATP production, in agreement with the changes observed in the metabolome and previous experiments in mouse embryonic fibroblasts, neurons, and isolated mitochondria (Holmstrom et al., 2013; Ludtmann et al., 2014). On the other hand, Nrf2 disruption decreased respiration, and the above respiration-associated parameters (Figure 2D, right panel).

In summary, these results support an essential role for Nrf2 in governing redox and intermediary metabolism in resting macrophages, and other cellular processes, such as the innate immune response.

### Nrf2 is a critical regulator of metabolism, mitochondrial adaptation, and innate immune pathways in inflammatory macrophages

To better understand the biological processes that Nrf2 regulates during an inflamed state, we performed an ORA on the significantly decreased (Figure 3A) and increased (Figure S5A; Table S4) proteins in Nrf2-KO macrophages stimulated with LPS. Enrichment analysis of positively regulated processes identified two functional clusters, that is, a large cluster that includes a plethora of intermediary metabolic pathways associated with carbohydrate, cofactor, and energy metabolism, and a smaller cluster for the cellular response to oxidative stress (Figure 3A). The most significantly enriched pathways included those involved in glycolysis and GSH metabolism, as well as hepoxilin biosynthesis (Figure S5C). In Keap1-KD positively regulated processes, a loosely interconnected functional cluster was observed with enrichment in processes associated with lipid metabolism, amino acid metabolism, and cofactor metabolism, while enrichment in regulators of reactive oxygen species (ROS), cell adhesion, and organic ion transport were also observed (Figure 3B; Table S5). The most significantly enriched processes included those related to FAO, carnitine metabolism, and GSH metabolism (Figure S5D). In contrast, we did not observe many significant processes in the proteome of LPS-treated Nrf2-KO macrophages with only one increase involving the negative regulation of macrophage chemotaxis (Figure S5A), whilst a large functional cluster was associated with the immune response, such as the regulation of T cell and leukocyte activation, adhesion, and proliferation, in the decreased targets of Keap1-KD macrophages (Figure S5B).

Like the resting state, LC-MS-based metabolomic analysis of WT, Nrf2-KO, and Keap1-KD macrophages stimulated with LPS (Figure 3C) revealed a significant increase in metabolites associated with the antioxidant response and bioenergetics. We also observed significant alterations in the abundance of several amino acids, with increased asparagine levels in Nrf2-KO, while tyrosine, leucine, and methionine were decreased, and arginine, lysine, and glycine were increased in the Keap1-KD cells. These findings support a central role for Nrf2 in governing macrophage intermediary metabolism, as predicted by our proteomic analyses (Figures 3A, 3B, S3C, and S3D). Likewise, analysis of extracellular acidification rates (ECAR) also confirmed a role for Nrf2 in promoting glycolysis in resting and activated macrophages (Figure 3D).

Interestingly, in addition to changes in metabolism, we also observed enrichment in mitochondrial fusion with Keap1-KD (Figure S5D), including the mitochondrial fusion proteins, Opa1, Mfn1, and Mfn2 (Figure S5F), and a significant increase in the mitochondrial fission factors, Mff and Mief2, with Nrf2-KO in activated macrophages (Figure S5F). Given the importance of mitochondrial physiology in governing cellular redox state, metabolism and bioenergetics, and the clear regulation of these processes by Nrf2, we hypothesized that Nrf2 status may modulate mitochondrial morphology. To explore this, we performed confocal microscopy analysis of mitochondrial morphology following immunofluorescence staining of the outer mitochondrial membrane (OMM) protein Tom20 (Figures 3E and S5E). Mitochondrial morphology was assigned as intermediate, fused/elongated, or fragmented (Tabara and Prudent, 2020). In the unstimulated state, mitochondria predominantly exhibited intermediate morphology across all three genotypes; however, a minority of Keap1-KD mitochondria exhibited fragmented or fused/elongated morphologies (Figure S5E). Interestingly, 24 h of LPS stimulation in WT macrophages caused a notable change, with 45% of cells displaying fused/elongated morphology, while the percentage of cells with intermediate morphology decreased from 95 to 55% (Figures 3E and S5E). The LPS-mediated change in mitochondrial morphology was even more striking in Keap1-KD cells, where the percentage of cells with intermediate mitochondria decreased from 75 to 25%, whereas the percentage of cells with fused/elongated morphology increased, from five- to 75% (Figures 3E and S5E). In contrast, LPS treatment of Nrf2-KO cells resulted in only a modest increase in fused/elongated mitochondria (10–25%), whereas the percentage of cells with intermediate mitochondria decreased from 90 to 75% (Figures 3E and S5E). Together, these experiments illustrate that prolonged stimulation of macrophages with LPS causes a switch in mitochondrial morphology, from intermediate to fused/elongated, which is enhanced by Nrf2 activation and suppressed by Nrf2 disruption. Therefore, Nrf2 represents a crucial factor governing redox and intermediary metabolism, mitochondrial adaptation, and innate immunity in macrophages upon encountering infectious stimuli.

### The Keap1 inhibitor, 4-octyl itaconate, promotes redox metabolism and inhibits the type I interferon response in inflammatory macrophages

During LPS stimulation, macrophages undergo profound metabolic changes, engaging aerobic glycolysis and suppressing OXPHOS (Ryan et al., 2019; Ryan and O'Neill, 2020). Importantly, several mitochondrial metabolites, including succinate, fumarate, and itaconate accumulate and act as signals to regulate macrophage effector functions (Mills et al., 2018; Ryan et al., 2019). Our previous work demonstrated that a lipophilic cell-permeable derivative of itaconate, 4-OI (Figure S1A), is a robust Nrf2 activator and anti-inflammatory compound (Mills et al., 2018). 4-OI activates Nrf2 via the alkylation of key cysteines on Keap1, and this is, at least in part, responsible for its anti-inflammatory effects (Figure S2E). In the same study, we found that 4-OI inhibited IFN-β production and the expression of IFN-inducible targets, but the role of Nrf2 was unclear. Therefore, we performed proteomic analysis of LPS-stimulated WT and Nrf2-KO macrophages that had been pre-treated with 4-OI for 3 h prior to LPS exposure to determine to what extent Nrf2 was involved in mediating the remodeling of the macrophage proteome upon the treatment of 4-OI (Figures S6A and S6B). Indeed, we observed significant changes in the proteome of activated macrophages treated with 4-OI in both genotypes; however, the impact was far more pronounced in WT cells (Figures S6A and S6B). We also confirmed a significant enrichment for Nrf2 in WT cells treated with 4-OI (Figure S6C).

**Figure 4:**
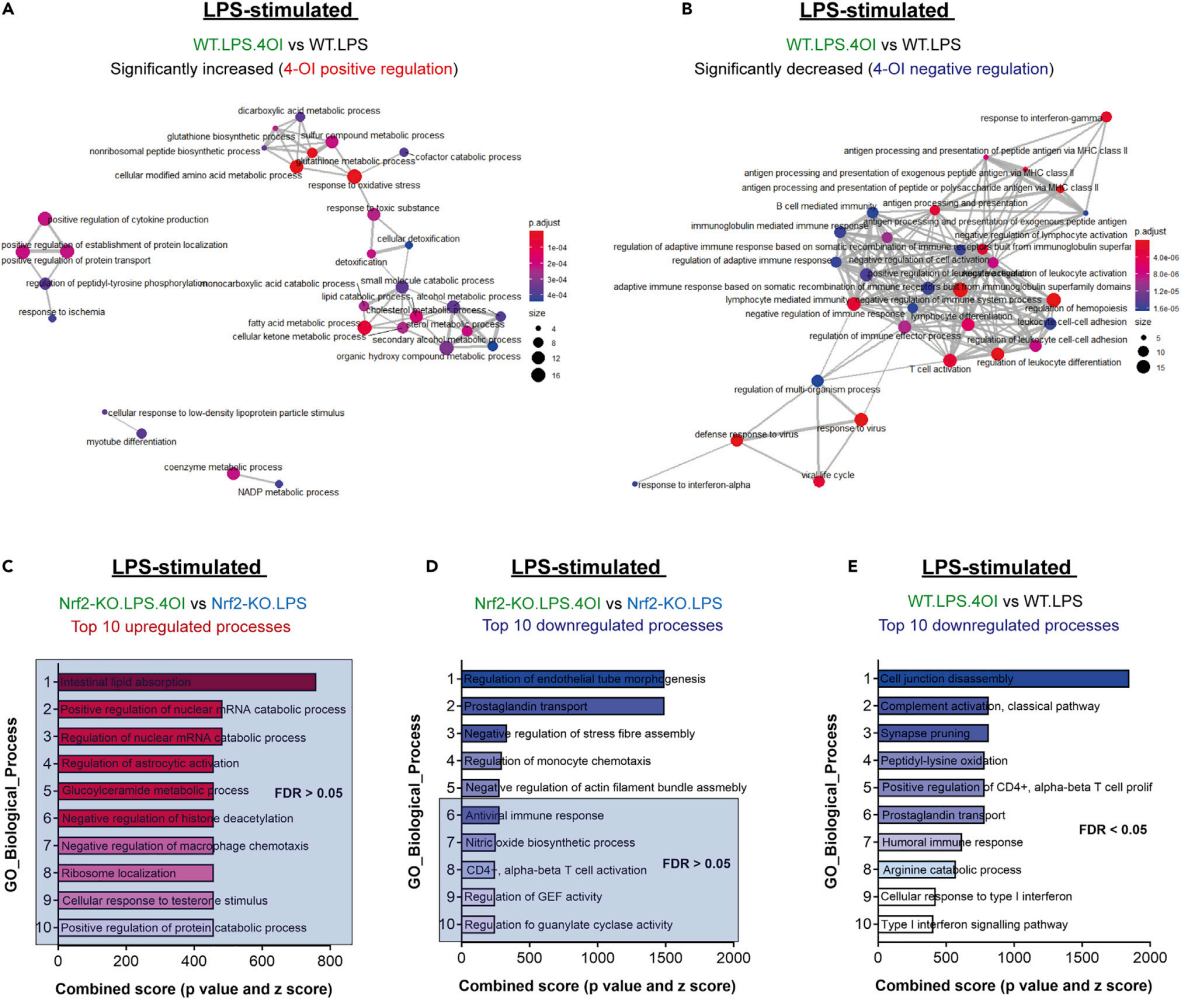
4-OI regulates redox metabolism and suppresses type I interferon response in an Nrf2-dependent manner (A) Enrichment map of GO: biological processes of 4-OI positively regulated targets (WT). (B) Enrichment map of GO: biological processes of 4-OI negatively regulated targets (WT) (A-B) ORA by clusterProfiler, FDR correction by Bonferroni test. (C) Enrichment of GO: biological processes of 4-OI positively regulated targets (Nrf2-KO). (D) Enrichment of GO: biological processes of 4-OI negatively regulated targets (Nrf2-KO). (E) Enrichment of GO: biological processes of 4-OI negatively regulated targets (WT). (A-E) 4-OI used at 125 μM (C-E) ORA by Enrichr and FDR correction by Bonferroni test. Top processes ranked according to combined enrichment score (p value and *Z* score).

To understand what biological processes 4-OI regulates, we performed ORA on the significant changes and found that in WT macrophages 4-OI regulates four functional clusters (Figure 4A; Table S6). Unsurprisingly, an enrichment for redox metabolism, detoxification, and lipid metabolism was identified, while an increase in positive regulators of cytokine production also emerged (Figures 4A and S6C). 4-OI significantly decreased type I IFN response proteins (Figures 4B, 4E, and S6D) and positive regulators of leukocyte activation, such as Nos2 (Figure S6A), consistent with its reported anti-inflammatory role (Mills et al., 2018) and the linear correlation (spanning six orders of magnitude of concentration) between the ability of structurally diverse Nrf2 activators to induce the Nrf2 target Nqo1 and to inhibit Nos2 (Dinkova-Kostova et al., 2005; Liu et al., 2008). Strikingly, in Nrf2-KO macrophages, there were no biological processes that reached significance and 4-OI lost its ability to regulate both redox metabolism and certain immune response effectors (Figure 4C; Table S7). Of note, 4-OI was less able to suppress type I IFN response proteins, such as IFN-induced protein with tetratricopeptide repeats 2 (Ifit2), and other IFN associated effector proteins in Nrf2-KO macrophages (Figures 4D and S6D). 4-OI was also found to decrease prostaglandin transporters in a Nrf2-independent manner (Figures 4D and 4E), which is consistent with a recent report demonstrating inhibition of prostaglandin synthesis and release in macrophages (Diskin et al., 2021). This highlights an important role for Nrf2 in mediating certain, but not all aspects of the immunomodulatory capabilities of 4-OI.

### Pharmacologic or genetic Nrf2 activation inhibits the type I interferon response in inflammatory macrophages

**Figure 5:**
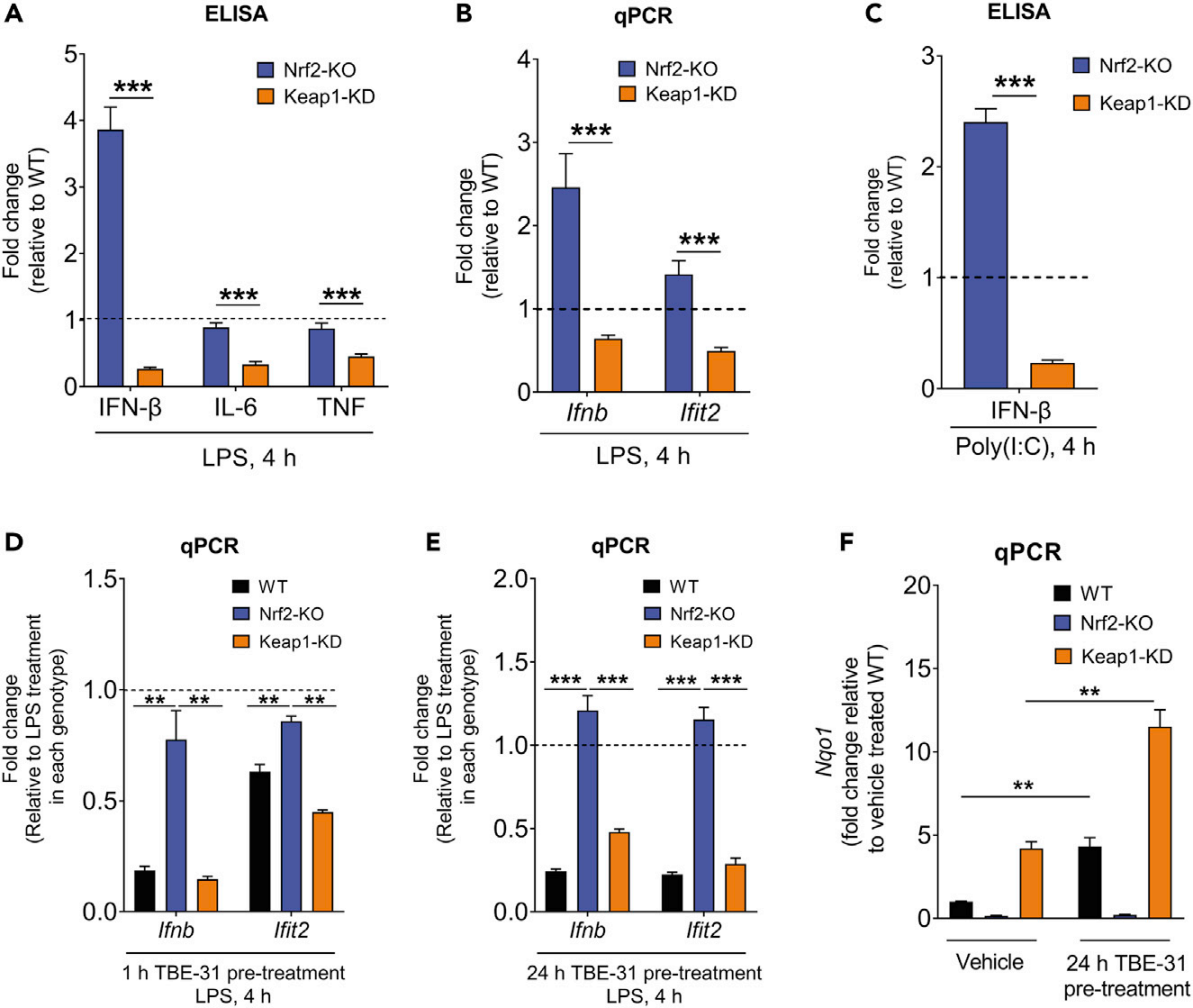
Nrf2 is an endogenous suppressor of IFN-β in inflammatory macrophages (A) IFN-β, IL-6, and TNF protein levels in LPS-stimulated Nrf2-KO and Keap1-KD compared to WT cells (n = 3 biological replicates). (B) Ifnb and Ifit2 mRNA levels in LPS-stimulated Nrf2-KO and Keap1-KD compared to WT cells (n = 5-6 biological replicates). (A-B) Data are mean ± SEM p value determined by unpaired t test, corrected for multiple comparisons by Holm-Sidak test. (C) IFN-β in Poly(I:C) stimulated Nrf2-KO and Keap1-KD compared to WT cells (n = 3 biological replicates). Data are mean ± SEM p value determined by two-tailed unpaired t test. (D) Ifnb and Ifit2 mRNA levels in WT, Nrf2-KO, and Keap1-KD cells that had been pre-treated for 1 h with the Nrf2 activator TBE-31 (30 nM) and stimulated with LPS (100 ng/mL) for a further 4 h (n = 3 biological replicates). (E) Ifnb and Ifit2 mRNA levels in WT, Nrf2-KO, and Keap1-KD cells that had been pre-treated for 24 h with the Nrf2 activator TBE-31 (20 nM) and stimulated with LPS (100 ng/mL) for a further 4 h (n = 2-3 biological replicates). (D-E) Data are mean ± SEM p value determined by one-way ANOVA, corrected for multiple comparisons by Tukey statistical test. (F) Nqo1 mRNA levels in WT, Nrf2-KO, and Keap1-KD cells that had been pre-treated for 24 h with the Nrf2 activator TBE-31 (20 nM) (n = 2-3 biological replicates). Data are mean ± SEM p value determined by a two-tailed unpaired t test. p <0.05∗; p <0.01∗∗; p <0.001∗∗∗.

To strengthen the evidence that Nrf2 activation has an inhibitory effect on the type I IFN response, we measured the production of IFN-β in LPS-stimulated BMDMs of the three genotypes. Strikingly, compared to WT, the protein levels of IFN-β were ∼4-fold higher in Nrf2-KO and ∼4-fold lower in Keap1-KD cells (Figure 5A). As expected, the protein levels of IL-6 and TNF were also lower in LPS-stimulated Keap1-KD BMDMs in comparison with their WT counterparts (Figure 5A). Notably, however, in contrast to the increase in IFN-β, Nrf2 disruption did not affect significantly the protein levels of IL-6 and TNF, in agreement with previous reports (Baardman et al., 2018; Bambouskova et al., 2018; Knatko et al., 2015; Kobayashi et al., 2016; McGuire et al., 2016; Mills et al., 2018). Further qPCR analysis showed that the effect of Nrf2 on IFN-β was at the transcriptional level (Figure 5B). Moreover, the expression of *Ifit2*, a target of IFN-β, was increased in Nrf2-KO and decreased in Keap1-KD in comparison with WT cells (Figure 5B). In addition, IFN-β levels were ∼2.5-fold higher in Nrf2-KO and ∼4-fold lower in Keap1-KD following the stimulation of TLR3 with the synthetic double-stranded RNA mimic, polyinosinic–polycytidylic acid sodium salt (Poly(I:C)) (Figure 5C). Similar to the genetic activation of Nrf2, pharmacologic activation by pre-treatment with the tricyclic cyanoenone TBE-31 (Figure S1B) for 1 h also led to the reduction of the mRNA levels for IFN-β and IFIT2 in LPS-stimulated WT cells; this effect was significantly diminished in Nrf2-KO and enhanced in Keap1-KD BMDMs (Figure 5D). The Nrf2-dependent inhibitory effect of TBE-31 on *Ifnb* expression was also observed following pre-treatment for 24 h (Figure 5E), which resulted in Nrf2 activation in WT cells comparable to that of the Keap1 knockdown, as evidenced by the expression of *Nqo1* (Figure 5F). Together, these data strongly suggest that whereas Nrf2 activation is generally anti-inflammatory, its levels are particularly important for dampening the type I IFN response.

This conclusion is further supported by the increase in NQO1 mRNA levels (Figure S7A) and lower mRNA levels for the chemokine CXCL10 (Figure S7B), a downstream target of the type I 10.13039/501100007072IFN signaling pathway, when murine RAW 264.7 macrophage-like monocytes were treated with the pharmacologic Nrf2 activators the isothiocyanate sulforaphane (Figure S1C) or the pentacyclic cyanoenone CDDO (Figure S1D). Sulforaphane also decreased the levels of IFNB and CXCL10, while increasing NQO1, following the transfection of differentiated human THP1 cells with the activator of stimulator of IFN genes (STING) 2′3′-cGAMP, which is produced from ATP and GTP in response to the detection of cytoplasmic DNA, such as during viral infection (Figure S7C). Conversely, a knockdown of *NFE2L2* decreased the expression of NQO1 and increased the expression of CXCL10 (Figure S7D).

## Discussion

The cytoprotective Keap1-Nrf2 axis regulates the expression of networks of genes encoding proteins at the interface between redox and intermediate metabolism, allowing adaptation and survival under various stress conditions (Hayes and Dinkova-Kostova, 2014; Yamamoto et al., 2018). The downstream targets of Nrf2 have a multitude of protective functions and, via their diverse detoxification, antioxidant and anti-inflammatory actions, protect against the damaging and immunotoxic effects of environmental pollutants (Suzuki et al., 2020). Thus, intervention studies in humans employing a pharmacological Nrf2 activation strategy have demonstrated accelerated detoxication of the air pollutant benzene; in this context, Nrf2 activation is expected to reduce the long-term health risks associated with unavoidable exposures to environmental pollution (Egner et al., 2014). In addition, by its anti-inflammatory actions, which are consistently being observed in animals and humans (Liu et al., 2020), Nrf2 activation prevents prolonged, chronic inflammation and potential tissue damage and health deterioration.

Interestingly, the protein and mRNA levels for IL1β, IL6, and TNF are not higher in LPS-stimulated Nrf2-knockout BMDM cells in comparison with their WT counterparts, in agreement with our previous observations in cutaneous tissue of Nrf2-knockout and WT mice following exposure to solar-simulated UV radiation, even though the expression of these cytokines is suppressed in UV-irradiated skin of Keap1-knockdown mice (Knatko et al., 2015). Other studies have also reported normal expression of TNF, IL6, and IL1β in the absence of Nrf2 (Baardman et al., 2018; Bambouskova et al., 2018; Kobayashi et al., 2016; McGuire et al., 2016; Mills et al., 2018). However, activation of Nrf2 in macrophages by pharmacologic or genetic means dampens inflammatory responses (Dayalan Naidu et al., 2018; Kobayashi et al., 2016; Mills et al., 2018). Macrophages from Keap1-mutant mice in which the critical cysteine 151 has been substituted with a serine, lose the ability to downregulate the expression of pro-inflammatory cytokines in response to pharmacological Nrf2 activators that are sensed through this cysteine (Dayalan Naidu et al., 2018). Interestingly, IFN-β levels were elevated by Nrf2 disruption, which suggests that Nrf2 may preferentially target the type I IFN system. Mechanistically, how Nrf2 represses *Ifnb* expression has yet to be determined. However, it’s possible that it may act as a transcriptional repressor by binding to non-ARE consensus sequences and directly interfere with RNA pol II recruitment, as previously reported for pro-inflammatory cytokines (Kobayashi et al., 2016). Together, these data suggest that the absence of Nrf2 may not enhance pro-inflammatory responses at the initial stages of inflammation, but that Nrf2 activation is anti-inflammatory.

Our high-resolution proteomics analysis revealed an unexpected role for Nrf2 as a critical regulator of not only redox but also intermediary metabolism, glycolysis, and mitochondrial respiration. Upon LPS stimulation, Nrf2 activation by Keap1 knockdown enhances the metabolic switch from oxidative phosphorylation to glycolysis. This is particularly important given the critical role of metabolic reprogramming for macrophage effector functions (Ryan and O'Neill, 2020). Unexpectedly, we also found that Nrf2 promoted fusion of mitochondrial networks in inflammatory macrophages, which may be due to changes in the abundance of mitochondrial fission/fusion proteins (Figures S5D and S5F), or alternatively, may be an indirect effect due to altered redox homeostasis, as recently reported in other contexts (Cvetko et al., 2021; Shutt et al., 2012). Because enhanced fusion protects mitochondrial integrity and maximizes the cellular oxidative capacity, we propose that in this way, Nrf2 maintains mitochondrial fitness whilst also supporting the necessary metabolic changes that allow mounting inflammatory responses during infection. Thus, the activation of Nrf2 has a striking capacity to govern mitochondrial physiology and could have implications for immunoregulatory events.

Notably, genetic or pharmacologic Nrf2 activation was found to suppress the type I interferon IFN-β and interferon-inducible protein IFIT2 (Figures 5A–5D). This finding is of interest due to the paradoxical role of Nrf2 as both an inhibitor of viral replication in certain contexts (Olagnier et al., 2020; Ordonez et al., 2021; Wyler et al., 2019) and a promoter in others (Olagnier et al., 2017). Other cellular stress response pathways, notably the PKR-induced integrated stress response (ISR) and Atf4 lead to a suppression of translation to prevent viral replication (Dauber and Wolff, 2009; Meurs et al., 1990). Given the known interplay of Atf4 and Nrf2 (Kasai et al., 2019), it is possible that Nrf2 may activate similar or unidentified responses to antagonize viral infection, even in the presence of a dampened type I IFN response and will require further investigation.

Finally, 4-OI has emerged as an anti-inflammatory compound with utility in various disease models via the activation of Nrf2 (Li et al., 2020; Liu et al., 2021; Olagnier et al., 2018, 2020; Zheng et al., 2020). Importantly, 4-OI represses IFN signaling both in response to viral stimuli and in cases of type I interferonopathies (Olagnier et al., 2018). Here, we confirm a central role for Nrf2 in mediating the immunomodulatory activity of 4-OI and other pharmacologic Nrf2 activators in inflammatory macrophages. Considering the interest in Nrf2 activators for the treatment of viral infection(s), care must be taken given the divergent response depending on the virus. This emphasizes the importance of future work in elucidating how Nrf2 activation modulates the response to specific bacterial and viral pathogens.

### Limitations of the study

The main limitation of our study is the fact that in our animal models, the genetic modifications of both *Nfe2l2* (encoding Nrf2) disruption and *Keap1* downregulation are global. Thus, we cannot exclude the possibility that systemic effects of these genetic modifications may indirectly affect the bone marrow cells, which were used to generate the BMDMs. To mitigate this potential risk, in all of our experiments, we have taken every precaution to ensure identical conditions during isolation, *ex vivo* differentiation, and experimental treatments of the corresponding BMDMs for each biological replicate of each genotype. Another limitation of our study is the fact that, in addition to Nrf2, Keap1 has other protein binding partners, which could be partly responsible for the observed changes due to the knockdown of Keap1. Keap1 is reported to downregulate NF-κB in cancer cells. However, our analysis did not yield enrichment for this TF. In contrast, we observed a decrease in pro-inflammatory cytokines and IFN with Keap1-KD, which indicates that Nrf2 is the primary target in macrophages. Overall, we believe that complementing this genetic approach by pharmacologic means for Nrf2 activation increases the robustness of the data and strengthens our conclusions.

## STAR★Methods

### Key resources table

**Table:** 

REAGENT or RESOURCE	SOURCE	IDENTIFIER
**Antibodies**		

Mouse/IgG2b anti-GAPDH	Proteintech	60004-1-Ig
Rat anti-Keap1, clone 144	Sigma-Aldrich	MABS514
Rabbit monoclonal anti-Nrf2	Cell Signaling Technology	12721
Rabbit monoclonal anti-Nqo1	Cell Signaling Technology	62262
Horseradish-peroxidase conjugated goat anti-rabbit secondary antibody	Cell Signaling Technology	7074
Polyclonal rabbit anti-TOM20 antibody	Proteintech	11802-1-AP
Goat anti-rabbit Alexa 569 antibody	Invitrogen	A11036
IRDye® 680RD Donkey anti-Mouse IgG Secondary Antibody	LI-COR	926–68072
IRDye® 680RD Goat anti-Rat IgG Secondary Antibody	LI-COR	926–68076
IRDye® 800CW Goat anti-Rabbit IgG Secondary Antibody	LI-COR	926–32211

**Chemicals, peptides, and recombinant proteins**		

Lipopolysaccharide (LPS) from *Escherichia coli* O111:B4	Sigma	L2630
Polyinosinic–polycytidylic acid sodium salt (Poly(I:C))	Sigma	P0913
2′3′-cGAMP	Invivogen	tlrl-nacga23-1
2′3′-cGAMP Control (Negative, 2′5′GpAp)	Invivogen	tlrl-nagpap
Lipofectamine™ RNAiMAX	Invitrogen	13778075
Lipofectamine™ 2000	Invitrogen	11668019
ON-TARGETplus human *NFE2L2* siRNA-SMARTpool	Dharmacon	L-003755-00–0005
siRNA negative control	Dharmacon	D-001810-10-50
4-octyl itaconate (4-OI)	Richard Hartley	Mills et al., (2018)
TBE-31	Tadashi Honda	Saito et al., 2013
2-cyano-3,10-dioxooleana-1,9(11)-dien-28-oic acid (CDDO)	Tadashi Honda	Honda et al., (1998)
*R,S*-Sulforaphane	LKT Labs	S8044
Phorbol 12-myristate 13-acetate	Sigma	P1585
Omniscript RT Kit	Qiagen	205113
PrimeScript™ RT Master Mix	Takara	RR036A
TaqMan™ Fast Advanced Master Mix	Applied Biosystems	4444557
Fast SYBR® Green Master Mix	Applied Biosystems	4385610

**Critical commercial assays**		

Mouse IFN beta ELISA Kit	Abcam	ab252363
Mouse IL-6 DuoSet ELISA	R&D systems	DY406
Mouse TNF-alpha DuoSet ELISA	R&D systems	DY410
TMTXplex™ Isobaric Mass Tagging Kit	Thermo Fisher Scientific	90406
RNeasy Mini Kit (50)	Qiagen	74104
High-Capacity cDNA Reverse Transcription Kit	Applied Biosystems	4368814

**Deposited data**		

MaxQuant proteome quantification output - TMT	PRIDE	PXD027737
Data-independent acquisition (DIA) proteomics	PRIDE	PXD030455
Differential expression results	This paper	Tables S1, S2, S3, S4, S5, S6 and S7

**Experimental models: Cell lines**		

Murine RAW 264.7 macrophage-like monocytes	ATCC	TIB-71
Human THP1 monocytes	Public Health England/ECACC	88081201

**Experimental models: Organisms/strains**		

*Mus musculus* C57BL/6 Wild type	In-house	Jackson Laboratory
*Mus musculus* C57BL/6 Nrf2^−/−^: Keap1^+/+^ (Nrf2-KO)	In-house	Higgins et al., (2009)
*Mus musculus* C57BL/6 Nrf2^−/−^: Keap1^fl/fl^ (Keap1-KD)	In-house	Taguchi et al., (2010)

**Oligonucleotides**		

Primers, see qPCR primers section in methods	This paper	M/A

**Software and algorithms**		

Metaboanalyst 5.0	Pang et al. (2021)	https://www.metaboanalyst.ca/
Bioconductor package limma via LFQ-analyst	Shah et al. (2020)	https://bioinformatics.erc.monash.edu/apps/LFQ-Analyst/
Enrichr	Kuleshov et al. (2016)	https://maayanlab.cloud/Enrichr/
Bioconductor package clusterProfiler 4.0	Wu et al., 2021	https://bioconductor.org/packages/release/bioc/html/clusterProfiler.html
Graphpad Prism 9.2.0	GraphPad	https://www.graphpad.com/scientific-software/prism/

**Taqman™ Gene expression assays and qPCR primers**		

*Nqo1* (mouse)	Applied Biosystems	Mm01253561_m1
*Cxcl10* (mouse)	Applied Biosystems	Mm00445235_m1
*Ifnb1* (mouse)	Applied Biosystems	Mm00439552_s1
*Ifit2* (mouse)	Applied Biosystems	Mm00492606_m1
*NQO1* (human)	Applied Biosystems	Hs02512143_s1
*CXCL10* (human)	Applied Biosystems	Hs00171042_m1
*IFNB1* (human)	Applied Biosystems	Hs01077958_s1
*NFE2L2* (human)	Applied Biosystems	Hs00975961_g1
*18S* (Human)	Applied Biosystems	Hs99999901_s1
*Il1b (mouse)* FW (5′-3′) TGGCAACTGTTCCTG RV (5′-3′) GGAAGCAGCCCTTCATCTTT	This paper	N/A
*Rps18 (mouse)* FW (5′-3′) TGGGAACTTCTCATCCCTTTGRV (5′-3′) GGATGTGAAGGATGGGAAGT	This paper	N/A
*Ifnb (mouse)* FW (5′-3′) CCCTATGGAGATGACGGAGA RV (5′-3′) CCCAGTGCTGGAGAAATTGT	This paper	N/A
*Ifit2 (mouse)* FW (5′-3′) CCTGGATCAAGAATGGGCTA RV (5′-3′) CATCCCACGATCCAGAAACT	This paper	N/A

### Resource availability

#### Lead contact

Further information and reasonable requests for resources and reagents should be directed to the lead contact: a.dinkovakostova@dundee.ac.uk

#### Materials availability

This study did not generate any unique materials.

### Experimental model and subject details

#### Animals

Mice were bred and maintained at the Medical School Resource Unit of the University of Dundee, with free access to water and food (pelleted RM1 diet from SDS Ltd., Witham, Essex, UK), on a 12-h light/12-h dark cycle, 35% humidity. Experimental design was in line with the 3Rs principles of replacement, reduction, and refinement (www.nc3rs.org.uk) and in accordance with the regulations described in the UK Animals (Scientific Procedures) Act 1986 and approved by the Welfare and Ethical use of Animals Committee of the University of Dundee. Wild-type, Nrf2^−/−^: Keap1^+/+^ (Nrf2-KO) (Higgins et al., 2009) and Nrf2^+/+^: Keap1^flox/flox^ (Keap1-KD) (Taguchi et al., 2010) mice were on the C57BL/6 genetic background. Both male and female mice were used, and the animals were always both age- and sex-matched within each individual experiment.

#### Generation and treatment of BMDMs

Mice were euthanized in a CO_2_ chamber and death was confirmed by cervical dislocation. Bone marrow cells were extracted from the leg bones and differentiated in DMEM (containing 10% fetal calf serum, 1% penicillin/streptomycin and 20% L929 supernatant) for 6 days at 37°C in a humidified 5% CO_2_ atmosphere, at which time they were counted and re-plated for experiments. Unless stated, 5 × 10^6^ BMDMs per milliliter were used in *in vitro* experiments. The LPS concentration used was 100  ng mL^−1^ (Sigma) and 4-OI (125 μM), synthesized as described (Mills et al., 2018), was administered to the cell culture medium 3 h before LPS was added, and was not removed during the subsequent 24 h of LPS stimulation. TBE-31 (used at 20 nM or 30 nM) was synthesized as described (Saito et al., 2013).

#### Cell lines and treatments

Murine macrophage-like RAW 264.7 cells were cultured at 37°C and 5% CO_2_ in DMEM containing 10% heat-inactivated fetal calf serum. For experiments, RAW 264.7 cells (3 × 10^5^ cells per well) were plated in 6-well plates, grown for 24 h, and exposed to sulforaphane (LKT Labs), CDDO (Honda et al., 1998), or vehicle (0.1% acetonitrile) for a further 24 h, and harvested for gene expression analysis of *Nqo1* and *Cxcl10* using quantitative real-time PCR (TaqMan^TM^) with *18S* as housekeeping gene. Human acute monocytic leukemia THP1 cells were grown in suspension at 37°C and 5% CO_2_ in RPMI supplemented with 10% heat-inactivated fetal calf serum. THP1 cells (4 × 10^5^ cells per well) in 6-well plates were differentiated into adherent macrophages by stimulation with 100 nM phorbol 12-myristate 13-acetate (PMA, Sigma-Aldrich) for 24 h, after which the medium was replaced with fresh medium without PMA and the cells were grown for a further 24 h. The cells were then exposed to sulforaphane or vehicle (0.1% acetonitrile) for a further 24 h, and subsequently transfected with 2′3′-cGAMP or 2′5′-GpAp (2′3′-cGAMP control) using Lipofectamine 2000 (Invitrogen). The cells were harvested 6 h post-transfection for gene expression analysis using quantitative real-time PCR with *18S* as housekeeping gene. To knockdown *NFE2L2*, THP1 cells were plated in medium containing PMA. On the next day, the medium was replaced with fresh medium without PMA, and the cells were grown for a further 24 h, following which they were transfected with si*NFE2L2* or negative control siRNA, and harvested 48 h post-transfection. The expression of *NFE2L2*, *NQO1* and *CXCL10* was determined by real-time PCR (TaqMan^TM^) with *18S* as housekeeping gene.

### Method details

#### Flow cytometry

BMDM cells (2 ×10^5^), isolated and differentiated as described above, were washed with ice-cold DPBS containing 2% fetal calf serum (Flow Buffer), re-suspended in 300 μL of the Flow Buffer containing 0.2 μg/mL 4′,6-diamidino-2-phenylindole (DAPI), acquired on BD LSRFortessa Cell Analyzer and analyzed on FlowJo software. Three biologically independent samples were analyzed.

#### Western blotting

Following the respective treatments, BMDM cells (5 × 10^5^) grown in 12-well plates were washed twice with PBS before lysing in SDS lysis buffer [50 mM Tris pH 6.8, 10% glycerol (v/v), 2% SDS (w/v) and 0.001% (w/v) Bromophenol Blue]. The samples were sonicated for 30 s at 20% amplitude before measuring the protein content using the BCA assay (Pierce). Lysates (15 μg total protein) were loaded onto 20-well 4–12% Bis-Tris NuPAGE gel (Thermo) and the proteins were separated by electrophoresis using MOPS buffer (Thermo). Separated proteins were transferred onto 0.45-μm premium nitrocellulose membranes (Amersham) by wet transfer (Biorad). Membranes were blocked in 5% (w/v) non-fat milk (Marvel) dissolved in PBS-0.1% (v/v) Tween 20 (PBST) (Milk-PBST) for 1 h. Immunoblotting was performed using primary antibodies generated against GAPDH (1:20000, Proteintech 60004-1-Ig), Keap1 (1:2000, Millipore MABS514), Nrf2 and Nqo1 (1:1000, Cell Signaling #12721, #62262), all of which were diluted in Milk-PBST. The membranes were incubated with primary antibodies overnight at 4°C or for 1 h at room temperature (RT) for GAPDH. Subsequently, the membranes were washed with PBST for 30 min before incubating with either the fluorescently conjugated secondary antibodies (1:20000) (LI-COR) or horseradish-peroxidase conjugated goat anti-rabbit secondary antibody for the Nrf2 blot (1:5000, #7074) (Cell Signaling) for 1 h at RT. Following secondary antibody incubation, the blots were washed for 30 min with PBST before visualizing the proteins by scanning the blots with the Odyssey CLx imager (LI-COR) or detecting the chemiluminescence signal using an X-ray film (Amersham).

#### RNA extraction and real-time quantitative (qPCR)

BMDMs were plated onto either 12-well plates (5 × 10^5^ cells per well) or 6-well plates (1 × 10^6^ per well), left to adhere overnight and treated as indicated. At experimental endpoint, cells were washed in PBS and then RNA was extracted using RNeasy kit (Qiagen) following the manufacturer’s instructions. RNA was eluted in water and then quantified using a Nanodrop (ThermoFisher Scientific). RNA (1 μg) was reverse-transcribed using High capacity cDNA Reverse Transcription kit (Applied Biosystems) or 500 ng of RNA was reverse-transcribed using PrimeScript™ RT Master Mix (Takara). For real-time qPCR, cDNA was run using Fast SYBR® Green Master Mix (Applied Biosystems) or TaqMan™ Fast Advanced Master Mix (Applied Biosystems) according to manufacturer’s instructions. Primers were either designed for the genes of interest using primer-BLAST, or pre-designed Taqman™ Gene Expression Assays were used (see Key Resource Table). *Rps18* or *18S* was used as the endogenous control. qPCR experiments were run on a 7900 HT Fast Real-Time PCR System or Quantstudio seven Flex Real-Time PCR System (Applied Biosystems).

#### TMT-based proteomic analysis

Sample preparation, tandem mass tag (TMT) labeling, protein digestion, fractionation and peptide LC-MS analysis were performed as described (Howden et al., 2019). Briefly, cell pellets were lysed in 400 μL lysis buffer [4% SDS50 mM tetraethylammonium bromide (pH 8.5) and 10 mM tris(2-carboxyethyl)phosphine hydrochloride], lysates were boiled and sonicated before alkylation with 20 mM iodoacetamide for 1 h at 22 °C in the dark. Proteins were digested with LysC and Trypsin, subjected to TMT labeling and the TMT-labelled samples were fractionated using high-pH reverse-phase chromatography. Fractions were dried, peptides (1 μg) dissolved in 5% formic acid and analyzed by LC-MS using an Orbitrap Fusion Tribrid mass spectrometer (Thermo Fisher Scientific) equipped with a Dionex ultra-high-pressure liquid chromatography system (RSLCnano).

#### TMT-based proteomics data processing

The TMT-labeled samples were collected and analyzed using MaxQuant (Cox and Mann, 2008; Tyanova et al., 2016) v. 1.6.2.10. The TMT channel mapping is shown in Table S9. The FDR threshold was set to 1% for each of the respective Peptide Spectrum Match (PSM) and Protein levels. The data were searched with the following parameters; type was set to Reporter ion MS3 with 10 plex TMT, stable modification of carbamidomethyl (C), variable modifications, oxidation (M), acetylation (protein N terminus), deamidation (NQ), with a two missed tryptic cleavages threshold. Minimum peptide length was set to six amino acids. Proteins and peptides were identified using Uniprot (SwissProt May 2018). Run parameters have been deposited to PRIDE (Perez-Riverol et al., 2019) along with the full MaxQuant quantification output (PXD027737). All corrected TMT reporter intensities were normalized and quantified to obtain protein copy number using the proteomic ruler method (Wisniewski et al., 2014) as described in (Howden et al., 2019).

#### DIA-based proteomic analysis

In this label-free method, 1.5 μg peptide was analyzed per sample. Samples were injected onto a nanoscale C18 reverse-phase chromatography system (UltiMate 3000 RSLC nano, Thermo Scientific) then electrosprayed into an Orbitrap Exploris 480 Mass Spectrometer (Thermo Scientific). For liquid chromatography buffers were as follows: buffer A (0.1% formic acid in Milli-Q water (v/v)) and buffer B (80% acetonitrile and 0.1% formic acid in Milli-Q water (v/v). Sample were loaded at 10 μL/min onto a trap column (100 μm × 2 cm, PepMap nanoViper C18 column, 5 μm, 100 Å, Thermo Scientific) equilibrated in 0.1% trifluoroacetic acid (TFA). The trap column was washed for 3 min at the same flow rate with 0.1% TFA then switched in-line with a Thermo Scientific, resolving C18 column (75 μm × 50 cm, PepMap RSLC C18 column, 2 μm, 100 Å). The peptides were eluted from the column at a constant flow rate of 300 nL/min with a linear gradient from 3% buffer B to 6% buffer B in 5 min, then from 6% buffer B to 35% buffer B in 115 min, and finally to 80% buffer B within 7 min. The column was then washed with 80% buffer B for 4 min and re-equilibrated in 35% buffer B for 5 min. Two blanks were run between each sample to reduce carry-over. The column was kept at a constant temperature of 40°C.

The data were acquired using an easy spray source operated in positive mode with spray voltage at 2.650 kV, and the ion transfer tube temperature at 250°C. The MS was operated in DIA mode. A scan cycle comprised a full MS scan (m/z range from 350–-1650), with RF lens at 40%, AGC target set to custom, normalized AGC target at 300, maximum injection time mode set to custom, maximum injection time at 20 ms and source fragmentation disabled. MS survey scan was followed by MS/MS DIA scan events using the following parameters: multiplex ions set to false, collision energy mode set to stepped, collision energy type set to normalized, HCD collision energies set to 25.5, 27 and 30, orbitrap resolution 30000, first mass 200, RF lens 40, AGC target set to custom, normalized AGC target 3000, maximum injection time 55 ms.

#### DIA-based proteomic data processing

The DIA data were processed with Spectronaut () version 15. It was searched against the murine SwissProt database in a library free mode using directDIA. The Qvalue was set to 1% at both the precursor and protein levels. The enzyme rule was set to ‘Trypsin/P’ and variable modification set to Acytel (N-term), Deamidation (NQ) and Oxidation (M). The quantification at the Major Group Quantity was set to the ‘Sum peptide quantity’ and the Minor Group Quantity was to ‘Sum precursor quantity’. The Top N feature for both Major and Minor groups were disabled. The full parameters can be seen within the Spectronaut file in the PRIDE submission (PXD030455).

#### LC-MS metabolomics

##### Steady-state metabolomics

For steady-state metabolomics, 5 × 10^5^ cells were plated the day before onto 12-well plates (5 technical replicates from three biological replicates) and extracted at the appropriate experimental endpoint (24 h timepoint). Prior to metabolite extraction, cells were counted using a hemocytometer using a separate counting plate prepared in parallel and treated exactly like the experimental plate. At the experimental endpoint, the media was aspirated off and the cells were washed at room temperature with PBS and placed on a cold bath with dry ice. Metabolite extraction buffer (MES) was added to each well following the proportion 1 × 10^6^ cells/0.5 mL of buffer. After 10 min, the plates were stored at −80°C freezer and kept overnight. The following day, the extracts were scraped and mixed at 4°C for 15 min in a thermomixer at 2000 rpm. After final centrifugation at max speed for 20 min at 4°C, the supernatants were transferred into labeled LC-MS vials.

##### Liquid chromatography coupled to mass spectrometry (LC-MS) analysis

HILIC chromatographic separation of metabolites was achieved using a Millipore Sequant ZIC-pHILIC analytical column (5 μm, 2.1 × 150 mm) equipped with a 2.1 × 20 mm guard column (both 5 mm particle size) with a binary solvent system. Solvent A was 20 mM ammonium carbonate, 0.05% ammonium hydroxide; Solvent B was acetonitrile. The column oven and autosampler tray were held at 40 and 4°C, respectively. The chromatographic gradient was run at a flow rate of 0.200 mL/min as follows: 0–2 min: 80% B; 2–17 min: linear gradient from 80% B to 20% B; 17–17.1 min: linear gradient from 20% B to 80% B; 17.1–22.5 min: hold at 80% B. Samples were randomized and analyzed with LC-MS in a blinded manner with an injection volume was 5 μL. Pooled samples were generated from an equal mixture of all individual samples and analyzed interspersed at regular intervals within sample sequence as a quality control.

Metabolites were measured with a Thermo Scientific Q Exactive Hybrid Quadrupole-Orbitrap Mass spectrometer (HRMS) coupled to a Dionex Ultimate 3000 UHPLC. The mass spectrometer was operated in full-scan, polarity-switching mode, with the spray voltage set to +4.5 kV/-3.5 kV, the heated capillary held at 320°C, and the auxiliary gas heater held at 280°C. The sheath gas flow was set to 25 units, the auxiliary gas flow was set to 15 units, and the sweep gas flow was set to 0 unit. HRMS data acquisition was performed in a range of *m/z* = 70–900, with the resolution set at 70,000, the AGC target at 1 × 10^6^, and the maximum injection time (Max IT) at 120 ms. Metabolite identities were confirmed using two parameters: (1) precursor ion m/z was matched within 5 ppm of theoretical mass predicted by the chemical formula; (2) the retention time of metabolites was within 5% of the retention time of a purified standard run with the same chromatographic method. The acquired spectra were analyzed using XCalibur Qual Browser and XCalibur Quan Browser software (Thermo Scientific) and the peak area for each detected metabolite was normalized against the total ion count (TIC) of that sample to correct any variations introduced from sample handling through instrument analysis. The normalized areas were used as variables for further statistical data analysis.

#### Oxygen consumption rate (OCR) and extracellular acidification rate (ECAR) measurements

Oxygen consumption rate (OCR) and extracellular acidification rate (ECAR) were measured using the real-time flux analyzer Seahorse XF24 (Agilent) according to a method modified from Van den Bossche et al. (Van den Bossche et al., 2015). In brief, 0.5 × 10^5^ cells were plated onto the instrument cell plate 27 h before the experiment in complete RPMI 1640 medium (Invitrogen) supplemented with 10% fetal calf serum, 2 mM L-glutamine and 1 mM Na-pyruvate (5 replicate wells for each condition). Following adhesion, the cells were treated as indicated for 24 h. At the treatment endpoint, the cell culture medium was replaced with XF RPMI medium pH 7.4 (Agilent, 103576–100) supplemented with 2 mM glutamine prior to analysis. Cells were treated with 25 mM glucose, 1.5 μM oligomycin, 1.5 μM FCCP/1 mM Na-pyruvate and 2.5 μM antimycin A/1.25 μM rotenone to assess the respiration parameters.

#### Confocal microscopy

##### Mitochondrial morphology

BMDMs were fixed with 4% (w/v) PFA in PBS for 15 min, 37°C, 5% CO_2_ and then washed three times with PBS. Autofluorescence was quenched with 50 mM NH_4_Cl for 10 min at room temperature, followed by three washes with PBS. BMDMs were permeabilized with 0.1% (v/v) Triton X-100 in PBS for 10 min at room temperature. The permeabilized cells were then blocked with 10% FBS in PBS for 20 min at room temperature. BMDMs were incubated in rabbit anti-TOM20 antibody (Proteintech, 11802-1-AP) at 1:1000 dilution in 5% fetal calf serum in PBS for 2 h at room temperature followed by three washes in 5% fetal calf serum in PBS. Cells were then incubated in goat anti-rabbit Alexa 569 antibody (Invitrogen, A11036) at 1:1000 dilution in 5% fetal calf serum for 1 h at room temperature. BMDMs were washed three times with PBS and then stored in PBS at 4°C until imaging.

BMDMs were imaged using a 100x oil objective lens with 500 ms exposure time, 50% laser intensity using excitation/emission wavelengths 561/620–60 nm on an Andor Spinning Disk confocal microscope. Images were analyzed using Fiji ImageJ.

Mitochondrial morphology was assigned as intermediate, fused/elongated or fragmented and presented as mean % of all cells ± SEM. 75 cells were counted for each condition, for three mice.

### Quantification and statistical analysis

#### Statistical analysis

For metabolomics data, metaboanalyst 5.0 (Pang et al., 2021) was used to analyze, perform statistics and visualize the results. Autoscaling of features (metabolites) was used for heatmap generation. One-way ANOVA corrected for multiple comparisons by the Tukey statistical test was used and a p. Adjusted <0.05 was set as the cut-off. For proteomics data, protein copy number was converted to a log_2_ scale and biological replicates were grouped by experimental condition. Protein-wise linear models combined with empirical Bayes statistics were used for the differential expression analyses. The Bioconductor package limma was used to carry out the analysis using an R based online tool (Shah et al., 2020). Data were visualized using a Volcano plot, which shows the log_2_ fold change on the x axis and the adjusted p value on the y axis. The cut-offs for analysis were a log_2_FC of 0.5 and an FDR <0.05, determined using t statistics. Over-representation analysis (ORA) of significant changes were assessed using Enrichr (Kuleshov et al., 2016) and the Bioconductor package clusterProfiler 4.0 in R (version 3.6.1). Transcription factor (TF) enrichment used the ENCODE and ChEA databases and was presented using a Clustergram via Enrichr. The red diagonal bars represent the combined TF enrichment score (P value and *Z* score). Further information on this visualization method is available at (Kuleshov et al., 2016). Emapplots were generated using enrichplot package in R (version 3.6.1). Graphpad Prism 9.2.0 was used to calculate statistics in bar plots using appropriate statistical text depending on the data including one-way ANOVA, two-tailed unpaired t test and multiple t tests. Adjusted p values were assessed using appropriate correction methods, such as Tukey and Holm-Sidak tests. p <0.05∗; p <0.01∗∗; p < 0.001∗∗∗.

## Data Availability

This study did not generate any new codes. Proteomics data have been submitted to PRIDE with the identifiers PXD027737 (TMT) and PXD030455 (DIA). Any additional information required to reanalyze the data reported in this paper is available from the lead contact upon request.

## Supplemental information

Document S1. Figures S1–S7

Table S1. Nrf2-KO_vs_WT_Differential_Expression_TMT, related to Figure S1

Table S2. Keap1-KD_vs_WT_ Differential_Expression_TMT, related to Figure S1

Table S3. WT.LPS_vs_WT_Differential_Expression_TMT, related to Figure S1

Table S4. Nrf2-KO.LPS_vs_Nrf2-KO_Differential_Expression_TMT, related to Figure S1

Table S5. Keap1-KD.LPS_vs_Keap1-KD_Differential_Expression_TMT, related to Figure S1

Table S6. WT.LPS.4OI_vs_WT.LPS_Differential_Expression_TMT, related to Figure S4

Table S7. Nrf2-KO.LPS.4OI_vs_Nrf2-KO.LPS_Differential_Expression_TMT, related to Figure S4

Table S8. Metabolomics_WT_Nrf2-KO_Keap1-KD_with_or_without_LPS, related to Figure S2

Table S9. TMT channel mapping, Related to STAR Methods

## References

[bib1] Baardman J., Verberk S.G.S., Prange K.H.M., van Weeghel M., van der Velden S., Ryan D.G., Wust R.C.I., Neele A.E., Speijer D., Denis S.W. (2018). A defective pentose phosphate pathway reduces inflammatory macrophage responses during hypercholesterolemia. Cell Rep..

[bib2] Bambouskova M., Gorvel L., Lampropoulou V., Sergushichev A., Loginicheva E., Johnson K., Korenfeld D., Mathyer M.E., Kim H., Huang L.H. (2018). Electrophilic properties of itaconate and derivatives regulate the IkappaBzeta-ATF3 inflammatory axis. Nature.

[bib3] Cox J., Mann M. (2008). MaxQuant enables high peptide identification rates, individualized p.p.b.-range mass accuracies and proteome-wide protein quantification. Nat. Biotechnol..

[bib4] Cuadrado A., Pajares M., Benito C., Jimenez-Villegas J., Escoll M., Fernandez-Gines R., Yague A.J.G., Lastra D., Manda G., Rojo A.I., Dinkova-Kostova A.T. (2020). Can activation of NRF2 Be a strategy COVID-19?. Trends Pharmacological Sciences.

[bib5] Cvetko F., Caldwell S.T., Higgins M., Suzuki T., Yamamoto M., Prag H.A., Hartley R.C., Dinkova-Kostova A.T., Murphy M.P. (2021). Nrf2 is activated by disruption of mitochondrial thiol homeostasis but not by enhanced mitochondrial superoxide production. J. Biol. Chem..

[bib6] Dauber B., Wolff T. (2009). Activation of the antiviral kinase PKR and viral countermeasures. Viruses.

[bib7] Dayalan Naidu S., Muramatsu A., Saito R., Asami S., Honda T., Hosoya T., Itoh K., Yamamoto M., Suzuki T., Dinkova-Kostova A.T. (2018). C151 in KEAP1 is the main cysteine sensor for the cyanoenone class of NRF2 activators, irrespective of molecular size or shape. Scientific Rep..

[bib8] Dinkova-Kostova A.T., Liby K.T., Stephenson K.K., Holtzclaw W.D., Gao X., Suh N., Williams C., Risingsong R., Honda T., Gribble G.W. (2005). Extremely potent triterpenoid inducers of the phase 2 response: correlations of protection against oxidant and inflammatory stress. Proc. Natl. Acad. Sci. U S A.

[bib9] Diskin C., Zotta A., Corcoran S.E., Tyrrell V.J., Zaslona Z., O'Donnell V.B., O'Neill L.A.J. (2021). 4-Octyl-Itaconate and dimethyl fumarate inhibit COX2 expression and prostaglandin production in macrophages. J. Immunol..

[bib10] Egner P.A., Chen J.G., Zarth A.T., Ng D.K., Wang J.B., Kensler K.H., Jacobson L.P., Munoz A., Johnson J.L., Groopman J.D. (2014). Rapid and sustainable detoxication of airborne pollutants by broccoli sprout beverage: results of a randomized clinical trial in China. Cancer Prev. Res. (Phila).

[bib11] Harvey C.J., Thimmulappa R.K., Sethi S., Kong X., Yarmus L., Brown R.H., Feller-Kopman D., Wise R., Biswal S. (2011). Targeting Nrf2 signaling improves bacterial clearance by alveolar macrophages in patients with COPD and in a mouse model. Sci. Transl Med..

[bib12] Hayes J.D., Dinkova-Kostova A.T. (2014). The Nrf2 regulatory network provides an interface between redox and intermediary metabolism. Trends Biochem. Sci..

[bib13] Higgins L.G., Kelleher M.O., Eggleston I.M., Itoh K., Yamamoto M., Hayes J.D. (2009). Transcription factor Nrf2 mediates an adaptive response to sulforaphane that protects fibroblasts in vitro against the cytotoxic effects of electrophiles, peroxides and redox-cycling agents. Toxicol. Appl. Pharmacol..

[bib14] Holmstrom K.M., Baird L., Zhang Y., Hargreaves I., Chalasani A., Land J.M., Stanyer L., Yamamoto M., Dinkova-Kostova A.T., Abramov A.Y. (2013). Nrf2 impacts cellular bioenergetics by controlling substrate availability for mitochondrial respiration. Biol. Open..

[bib15] Honda T., Rounds B.V., Gribble G.W., Suh N., Wang Y., Sporn M.B. (1998). Design and synthesis of 2-cyano-3,12-dioxoolean-1,9-dien-28-oic acid, a novel and highly active inhibitor of nitric oxide production in mouse macrophages. Bioorg. Med. Chem. Lett..

[bib16] Howden A.J.M., Hukelmann J.L., Brenes A., Spinelli L., Sinclair L.V., Lamond A.I., Cantrell D.A. (2019). Quantitative analysis of T cell proteomes and environmental sensors during T cell differentiation. Nat. Immunol..

[bib17] Kasai S., Yamazaki H., Tanji K., Engler M.J., Matsumiya T., Itoh K. (2019). Role of the ISR-ATF4 pathway and its cross talk with Nrf2 in mitochondrial quality control. J. Clin. Biochem. Nutr..

[bib18] Knatko E.V., Ibbotson S.H., Zhang Y., Higgins M., Fahey J.W., Talalay P., Dawe R.S., Ferguson J., Huang J.T., Clarke R. (2015). Nrf2 activation protects against solar-simulated ultraviolet radiation in mice and humans. Cancer Prev. Res. (Phila).

[bib19] Knatko E.V., Tatham M.H., Zhang Y., Castro C., Higgins M., Dayalan Naidu S., Leonardi C., de la Vega L., Honda T., Griffin J.L. (2020). Downregulation of Keap1 confers features of a fasted metabolic state. iScience.

[bib20] Kobayashi E.H., Suzuki T., Funayama R., Nagashima T., Hayashi M., Sekine H., Tanaka N., Moriguchi T., Motohashi H., Nakayama K., Yamamoto M. (2016). Nrf2 suppresses macrophage inflammatory response by blocking proinflammatory cytokine transcription. Nat. Commun..

[bib21] Kuleshov M.V., Jones M.R., Rouillard A.D., Fernandez N.F., Duan Q., Wang Z., Koplev S., Jenkins S.L., Jagodnik K.M., Lachmann A. (2016). Enrichr: a comprehensive gene set enrichment analysis web server 2016 update. Nucleic Acids Res..

[bib22] Li Y., Chen X., Zhang H., Xiao J., Yang C., Chen W., Wei Z., Chen X., Liu J. (2020). 4-Octyl itaconate alleviates lipopolysaccharide-induced acute lung injury in mice by inhibiting oxidative stress and inflammation. Drug Des. Dev. Ther..

[bib23] Liu G., Wu Y., Jin S., Sun J., Wan B.B., Zhang J., Wang Y., Gao Z.Q., Chen D., Li S. (2021). Itaconate ameliorates methicillin-resistant Staphylococcus aureus-induced acute lung injury through the Nrf2/ARE pathway. Ann. Transl Med..

[bib24] Liu H., Dinkova-Kostova A.T., Talalay P. (2008). Coordinate regulation of enzyme markers for inflammation and for protection against oxidants and electrophiles. Proc. Natl. Acad. Sci. U S A.

[bib25] Liu H., Zimmerman A.W., Singh K., Connors S.L., Diggins E., Stephenson K.K., Dinkova-Kostova A.T., Fahey J.W. (2020). Biomarker exploration in human peripheral blood mononuclear cells for monitoring sulforaphane treatment responses in autism Spectrum disorder. Scientific Rep..

[bib26] Ludtmann M.H., Angelova P.R., Zhang Y., Abramov A.Y., Dinkova-Kostova A.T. (2014). Nrf2 affects the efficiency of mitochondrial fatty acid oxidation. Biochem. J..

[bib27] Maruyama A., Tsukamoto S., Nishikawa K., Yoshida A., Harada N., Motojima K., Ishii T., Nakane A., Yamamoto M., Itoh K. (2008). Nrf2 regulates the alternative first exons of CD36 in macrophages through specific antioxidant response elements. Arch. Biochem. Biophys..

[bib28] McGuire V.A., Ruiz-Zorrilla Diez T., Emmerich C.H., Strickson S., Ritorto M.S., Sutavani R.V., Weibeta A., Houslay K.F., Knebel A., Meakin P.J. (2016). Dimethyl fumarate blocks pro-inflammatory cytokine production via inhibition of TLR induced M1 and K63 ubiquitin chain formation. Scientific Rep..

[bib29] Meurs E., Chong K., Galabru J., Thomas N.S., Kerr I.M., Williams B.R., Hovanessian A.G. (1990). Molecular cloning and characterization of the human double-stranded RNA-activated protein kinase induced by interferon. Cell.

[bib30] Mills E.L., Ryan D.G., Prag H.A., Dikovskaya D., Menon D., Zaslona Z., Jedrychowski M.P., Costa A.S.H., Higgins M., Hams E. (2018). Itaconate is an anti-inflammatory metabolite that activates Nrf2 via alkylation of KEAP1. Nature.

[bib31] Olagnier D., Brandtoft A.M., Gunderstofte C., Villadsen N.L., Krapp C., Thielke A.L., Laustsen A., Peri S., Hansen A.L., Bonefeld L. (2018). Nrf2 negatively regulates STING indicating a link between antiviral sensing and metabolic reprogramming. Nat. Commun..

[bib32] Olagnier D., Farahani E., Thyrsted J., Blay-Cadanet J., Herengt A., Idorn M., Hait A., Hernaez B., Knudsen A., Iversen M.B. (2020). SARS-CoV2-mediated suppression of NRF2-signaling reveals potent antiviral and anti-inflammatory activity of 4-octyl-itaconate and dimethyl fumarate. Nat. Commun..

[bib33] Olagnier D., Lababidi R.R., Hadj S.B., Sze A., Liu Y., Naidu S.D., Ferrari M., Jiang Y., Chiang C., Beljanski V. (2017). Activation of Nrf2 signaling augments vesicular stomatitis virus oncolysis via autophagy-driven suppression of antiviral immunity. Mol. Ther..

[bib34] Ordonez A.A., Bullen C.K., Villabona-Rueda A.F., Thompson E.A., Turner M.L., Davis S.L., Komm O., Powell J.D., D'Alessio F.R., Yolken R.H. (2021). Sulforaphane exhibits in vitro and in vivo antiviral activity against pandemic SARS-CoV-2 and seasonal HCoV-OC43 coronaviruses. bioRxiv.

[bib35] Pang Z., Chong J., Zhou G., de Lima Morais D.A., Chang L., Barrette M., Gauthier C., Jacques P.E., Li S., Xia J. (2021). MetaboAnalyst 5.0: narrowing the gap between raw spectra and functional insights. Nucleic Acids Res..

[bib36] Perez-Riverol Y., Csordas A., Bai J.W., Bernal-Llinares M., Hewapathirana S., Kundu D.J., Inuganti A., Griss J., Mayer G., Eisenacher M. (2019). The PRIDE database and related tools and resources in 2019: improving support for quantification data. Nucleic Acids Res..

[bib37] Ryan D.G., Murphy M.P., Frezza C., Prag H.A., Chouchani E.T., O'Neill L.A., Mills E.L. (2019). Coupling Krebs cycle metabolites to signalling in immunity and cancer. Nat. Metab..

[bib38] Ryan D.G., O'Neill L.A.J. (2020). Krebs cycle reborn in macrophage immunometabolism. Annu. Rev. Immunol..

[bib39] Saddawi-Konefka R., Seelige R., Gross E.T., Levy E., Searles S.C., Washington A., Santosa E.K., Liu B., O'Sullivan T.E., Harismendy O., Bui J.D. (2016). Nrf2 induces IL-17d to mediate tumor and virus surveillance. Cell Rep..

[bib40] Saito Z.S., Takahashi M., Li W., Ojima I., Honda T. (2013). An improved synthesis of a hydroxymethyl tricyclic ketone from cyclohexanone, the key process for the synthesis of a highly potent anti-inflammatory and cytoprotective agent. Synthesis.

[bib41] Shah A.D., Goode R.J.A., Huang C., Powell D.R., Schittenhelm R.B. (2020). LFQ-analyst: an easy-to-use interactive web platform to analyze and visualize label-free proteomics data preprocessed with MaxQuant. J. Proteome Res..

[bib42] Shutt T., Geoffrion M., Milne R., McBride H.M. (2012). The intracellular redox state is a core determinant of mitochondrial fusion. EMBO Rep..

[bib43] Sun Q., Ye F., Liang H., Liu H., Li C., Lu R., Huang B., Zhao L., Tan W., Lai L. (2021). Bardoxolone and bardoxolone methyl, two Nrf2 activators in clinical trials, inhibit SARS-CoV-2 replication and its 3C-like protease. Signal. Transduct Target Ther..

[bib44] Suzuki T., Hidaka T., Kumagai Y., Yamamoto M. (2020). Environmental pollutants and the immune response. Nat. Immunol..

[bib45] Tabara L.C., Prudent J. (2020). The last wall of defense to prevent extreme and deleterious mitochondrial fusion. Embo J..

[bib46] Taguchi K., Maher J.M., Suzuki T., Kawatani Y., Motohashi H., Yamamoto M. (2010). Genetic analysis of cytoprotective functions supported by graded expression of Keap1. Mol. Cell Biol..

[bib47] Thimmulappa R.K., Scollick C., Traore K., Yates M., Trush M.A., Liby K.T., Sporn M.B., Yamamoto M., Kensler T.W., Biswal S. (2006). Nrf2-dependent protection from LPS induced inflammatory response and mortality by CDDO-Imidazolide. Biochem. Biophys. Res. Commun..

[bib48] Tyanova S., Temu T., Sinitcyn P., Carlson A., Hein M.Y., Geiger T., Mann M., Cox J. (2016). The Perseus computational platform for comprehensive analysis of (prote)omics data. Nat. Methods.

[bib49] Van den Bossche J., Baardman J., de Winther M.P. (2015). Metabolic characterization of polarized M1 and M2 bone marrow-derived macrophages using real-time extracellular flux analysis. J. Vis. Exp..

[bib50] Wisniewski J.R., Hein M.Y., Cox J., Mann M. (2014). A "proteomic ruler" for protein copy number and concentration estimation without spike-in standards. Mol. Cell Proteomics.

[bib51] Wyler E., Franke V., Menegatti J., Kocks C., Boltengagen A., Praktiknjo S., Walch-Ruckheim B., Bosse J., Rajewsky N., Grasser F. (2019). Single-cell RNA-sequencing of herpes simplex virus 1-infected cells connects NRF2 activation to an antiviral program. Nat. Commun..

[bib52] Yamamoto M., Kensler T.W., Motohashi H. (2018). The KEAP1-NRF2 system: a thiol-based sensor-effector apparatus for maintaining redox homeostasis. Physiol. Rev..

[bib53] Zheng Y., Chen Z., She C., Lin Y., Hong Y., Shi L., Zhang Y., Cao P., Xu X. (2020). Four-octyl itaconate activates Nrf2 cascade to protect osteoblasts from hydrogen peroxide-induced oxidative injury. Cell Death Dis..

