# Supplementary material for: Nrf2 activation reprograms macrophage intermediary metabolism and suppresses the type I interferon response
Source: iScience. 2022 Jan 30;25(2):103827. doi: 10.1016/j.isci.2022.103827 (PMC8844662; doi:10.1016/j.isci.2022.103827)
Supplement: Document S1. Figures S1–S7 [file mmc1.pdf]

iScience, Volume *25*

Supplemental information

Nrf2 activation reprograms macrophage intermediary metabolism and suppresses the type I interferon response

Dylan G. Ryan, Elena V. Knatko, Alva M. Casey, Jens L. Hukelmann, Sharadha Dayalan Naidu, Alejandro J. Brenes, Thanapon Ekkunagul, Christa Baker, Maureen Higgins, Laura Tronci, Efterpi Nikitopolou, Tadashi Honda, Richard C. Hartley, Luke A.J. O'Neill, Christian Frezza, Angus I. Lamond, Andrey Y. Abramov, J. Simon C. Arthur, Doreen A. Cantrell, Michael P. Murphy, and Albena T. Dinkova-Kostova

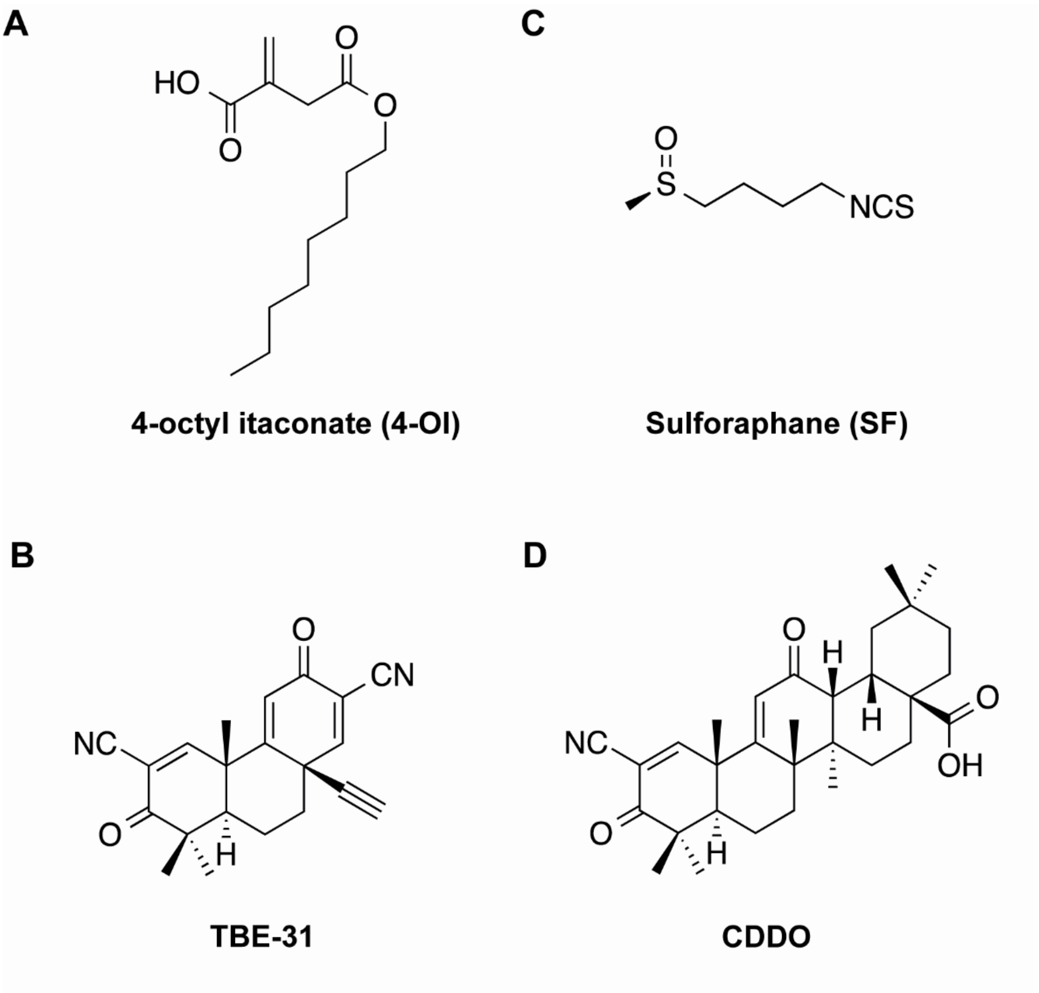

Figure S1 – Chemical structures of pharmacologic Nrf2 activators used in this study, related to STAR Methods

(A) 4-octyl itaconate (4-OI)

(B) Sulforaphane (SF)

(C) (±)-(4b*S*,8a*R*,10a*S*)-10a-ethynyl-4b,8,8-trimethyl-3,7-dioxo-3,4b,7,8,8a,9,10,10a-octahydrophenanthrene-2,6-dicarbonitrile (TBE-31)

(D) (+)-(4a*S*,6a*R*,6b*S*,8a*R*,12a*R*,14a*R*,14b*S*)-11-isocyano-2,2,6a,6b,9,9,12a-heptamethyl-10,14-dioxo-1,2,3,4,4a,5,6,6a,6b,7,8,8a,9,10,12a,14,14a,14b-octadecahydropicene-4a-carboxylic acid (CDDO)

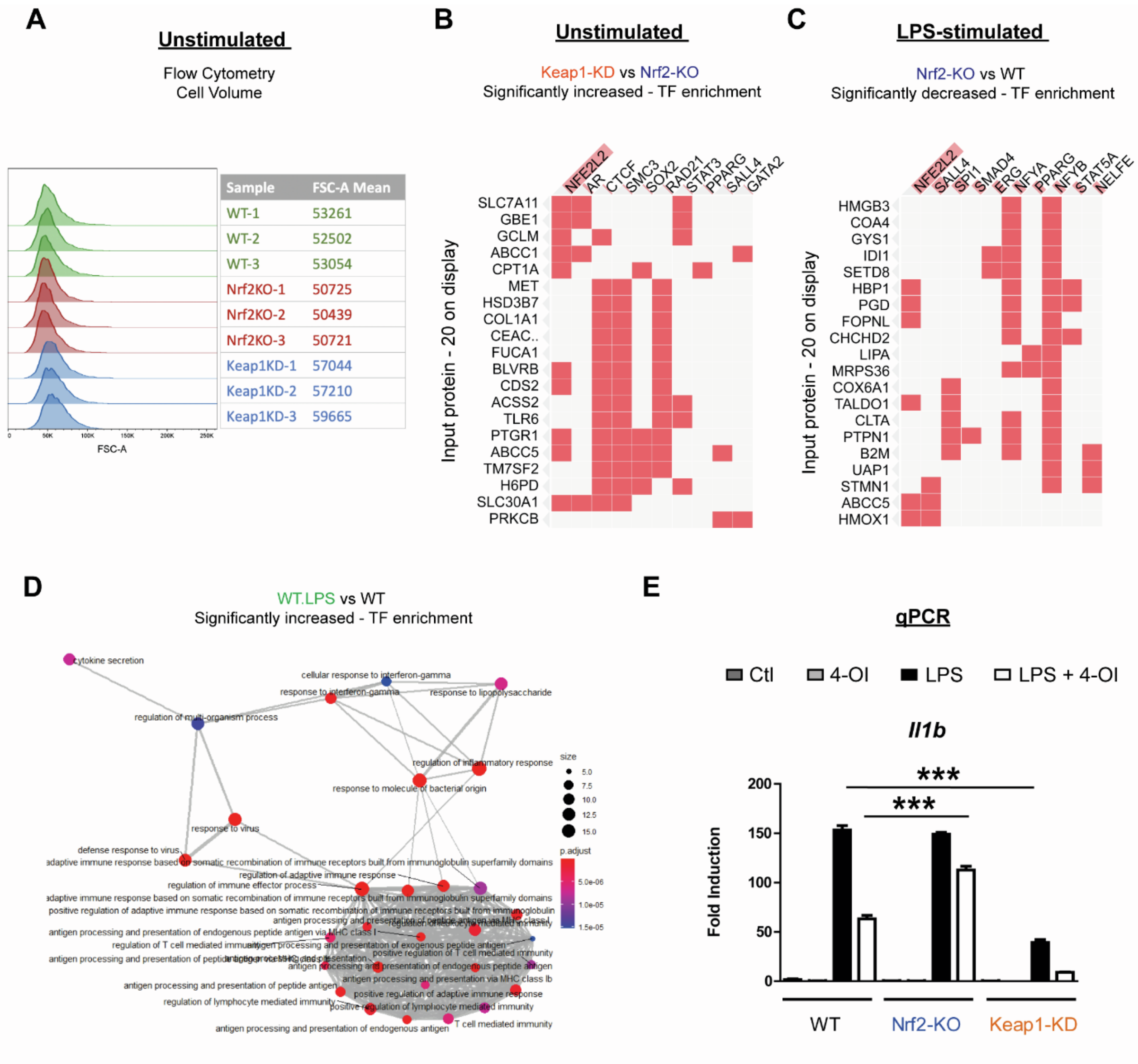

Figure S2 – LPS remodels macrophage proteome, related to Figure 1

(A) Flow cytometry analysis of cell size in unstimulated state

(B) TF enrichment of decreased targets in unstimulated Keap1-KD compared to Nrf2-KO

(C) TF enrichment of decreased targets in LPS stimulated Nrf2-KO compared to WT. ORA by Enricher. Top transcription factors ranked according to combined enrichment score (p value and Z score) using ENCODE and ChEA databases.

(D) Enrichment map of GO: biological processes of significantly increased proteins with LPS stimulation in WT

(E) qPCR (n = 3 biological replicates) validation of 4-OI (125 μM) inhibition of IL-1β **(A, E)** (n = 3 biological replicates). Data are mean ± SEM. p value determined by one-way ANOVA, corrected for multiple comparisons by Tukey statistical test. $p < 0.05$*; $p < 0.01$**; $p < 0.001$***. **(D)** ORA by clusterProfiler, FDR correction by Bonferroni test **(B-C)** (combined score – p value and z score).

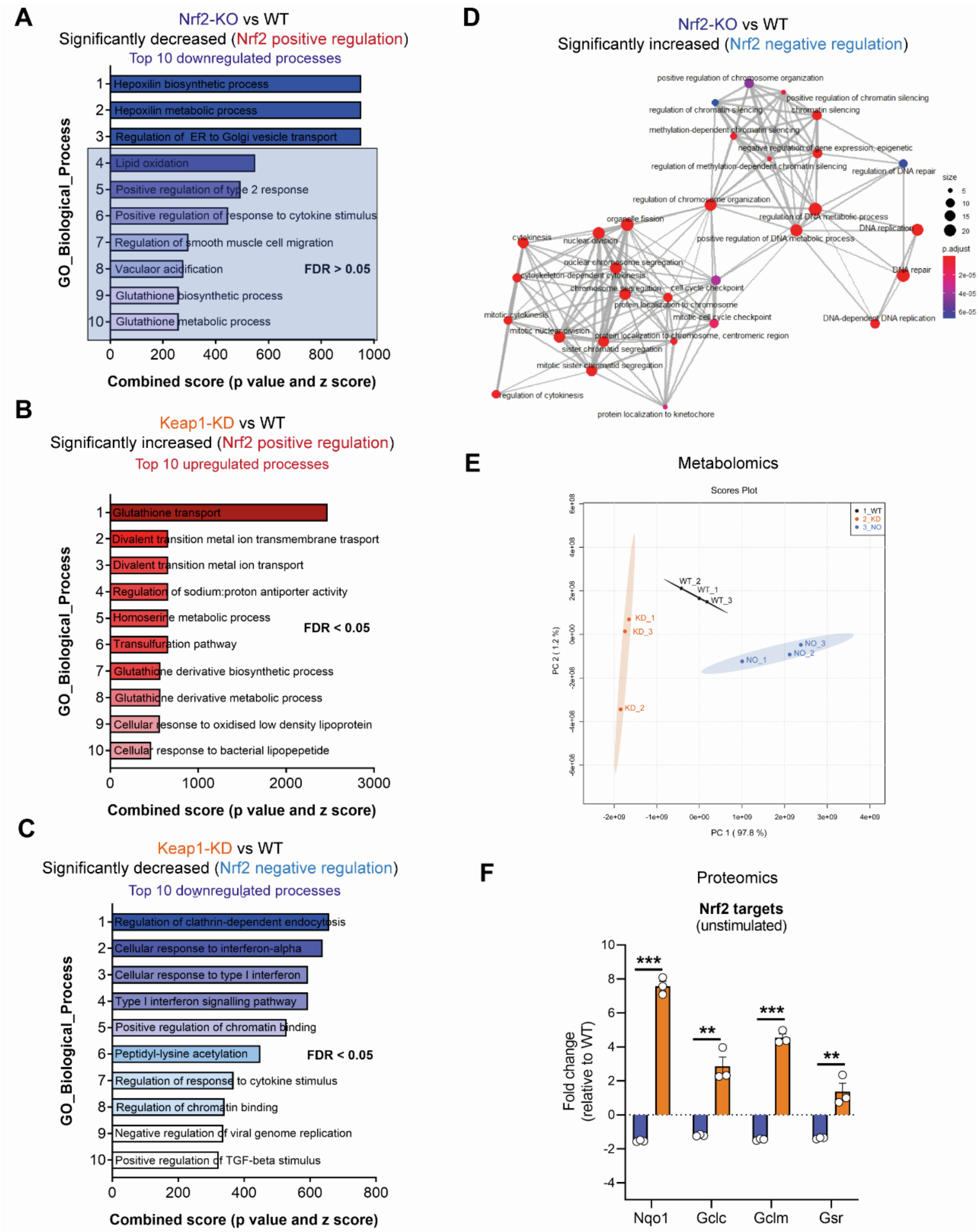

Figure S3 – Enrichment analysis of unstimulated macrophages, related to Figure 2

(A) Enrichment of GO: biological processes of Nrf2 positively regulated targets (Nrf2-KO vs WT)

(B) Enrichment of GO: biological processes of Nrf2 positively regulated targets (Keap1-KD vs WT)

(C) Enrichment of Reactome pathways of Nrf2 negatively regulated targets (Keap1-KD vs WT) **(A-C)** ORA by Enrichr and FDR correction by Bonferroni test. Top processes ranked according to combined enrichment score (p value and Z score).

(D) Enrichment map of GO: biological processes of Nrf2 negatively regulated targets (Nrf2-KO vs WT). ORA by clusterProfiler, FDR correction by Bonferroni test.

(E) PCA plot of metabolomics. Determined by Metaboanalyst 5.0.

(F) Fold change of prototypical Nrf2 targets (Nrf2-KO vs WT or Keap1-KD vs WT). Data are mean ± SEM. p value determined by multiple t tests - one per row, corrected for multiple comparisons by Holm-Sidak test. $p < 0.05$*; $p < 0.01$**; $p < 0.001$***.

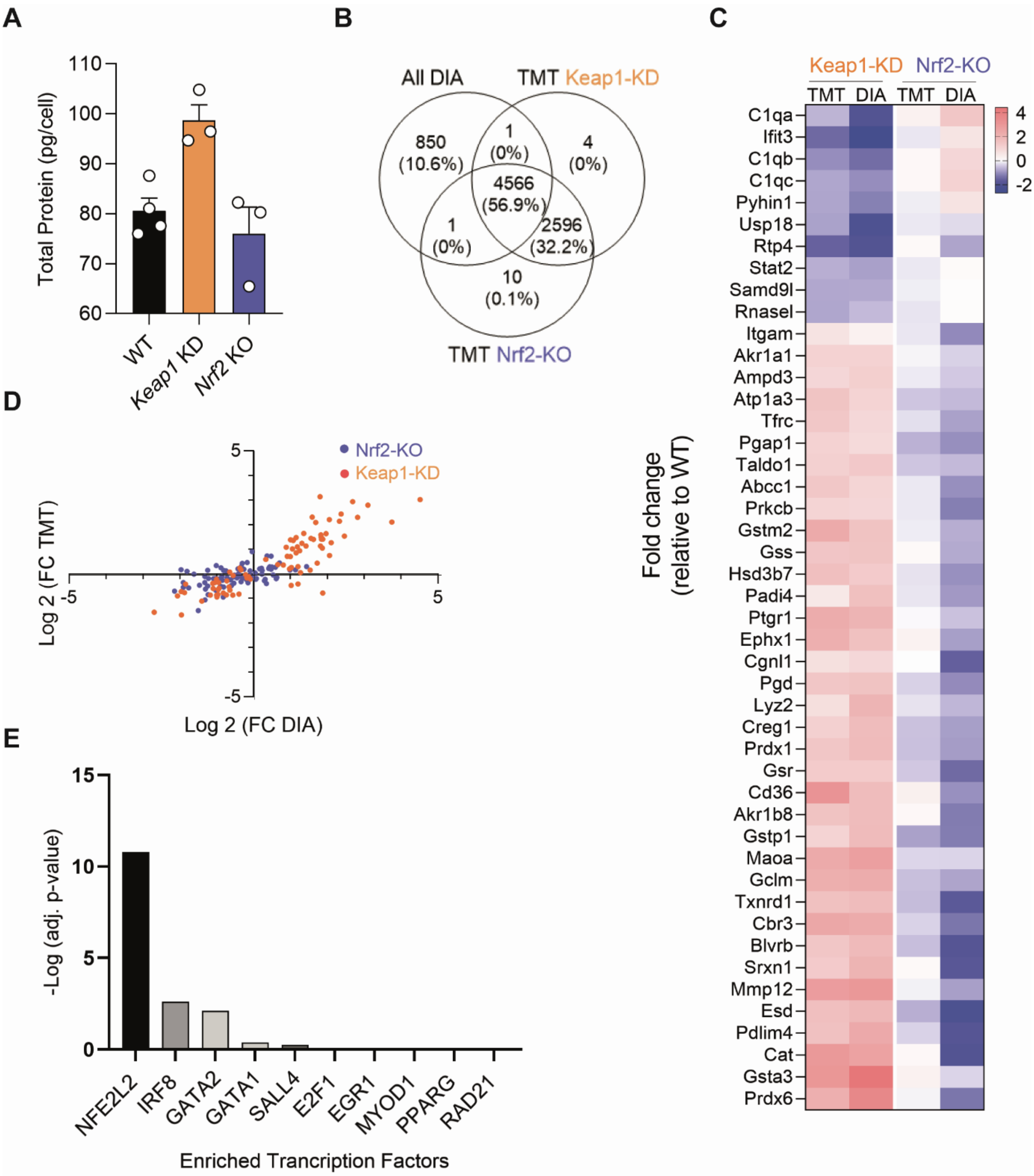

Figure S4 – Data-independent acquisition (DIA) proteomics, related to Figure 2

(A) Total protein content of WT, Nrf2-KO and Keap1-KD macrophages from DIA proteomics. Data are mean ± SEM.

(B) Venn diagram comparing protein hits from TMT and DIA datasets. DIA dataset includes proteins identified in both Nrf2-KO and Keap1-KD.

(C) Heatmap comparing significantly differentially regulated targets identified in TMT and DIA datasets. Data are fold change of Keap1-KD or Nrf2-KO relative to WT.

(D) Correlation plot comparing Log_2FC values of significantly differentially regulated targets identified in TMT and DIA datasets.

(E) TF enrichment of differentially regulated targets in Keap1-KD compared to WT. ORA by Enrichr. Top 10 targets ranked according to adjusted p value.

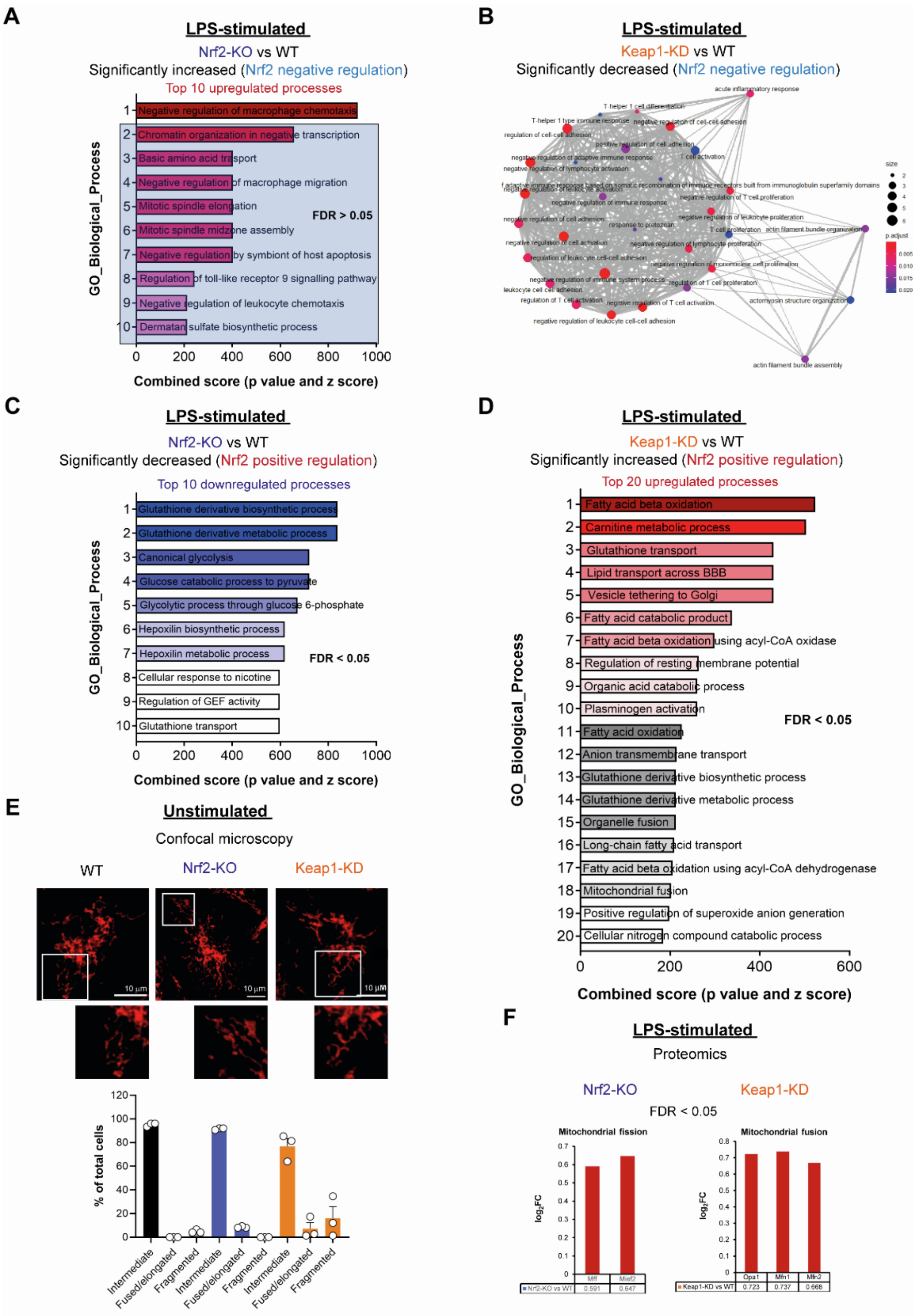

Figure S5 – Enrichment analysis of unstimulated macrophages, related to Figure 3

(A) Enrichment map of GO: biological processes of Nrf2 negatively regulated targets (Nrf2-KO vs WT)

(B) Enrichment map of GO: biological processes of Nrf2 negatively regulated targets (Keap1-KD vs WT) **(A-B)** ORA by clusterProfiler, FDR correction by Bonferroni test.

(C) Enrichment of GO: biological processes of Nrf2 positively regulated targets (Nrf2-KO vs WT)

(D) Enrichment of GO: biological processes of Nrf2 positively regulated targets (Keap1-KD vs WT)

(C-D) ORA by Enrichr and FDR correction by Bonferroni test. Top processes ranked according to combined enrichment score (p value and Z score).

(E) Confocal microscopy of mitochondrial morphology using Tom20 (images are representative, bar plot n = 3 biological replicates). Scale bar 10 μm. Data are mean ± SEM. p value determined by one-way ANOVA, corrected for multiple comparisons by Tukey statistical test.

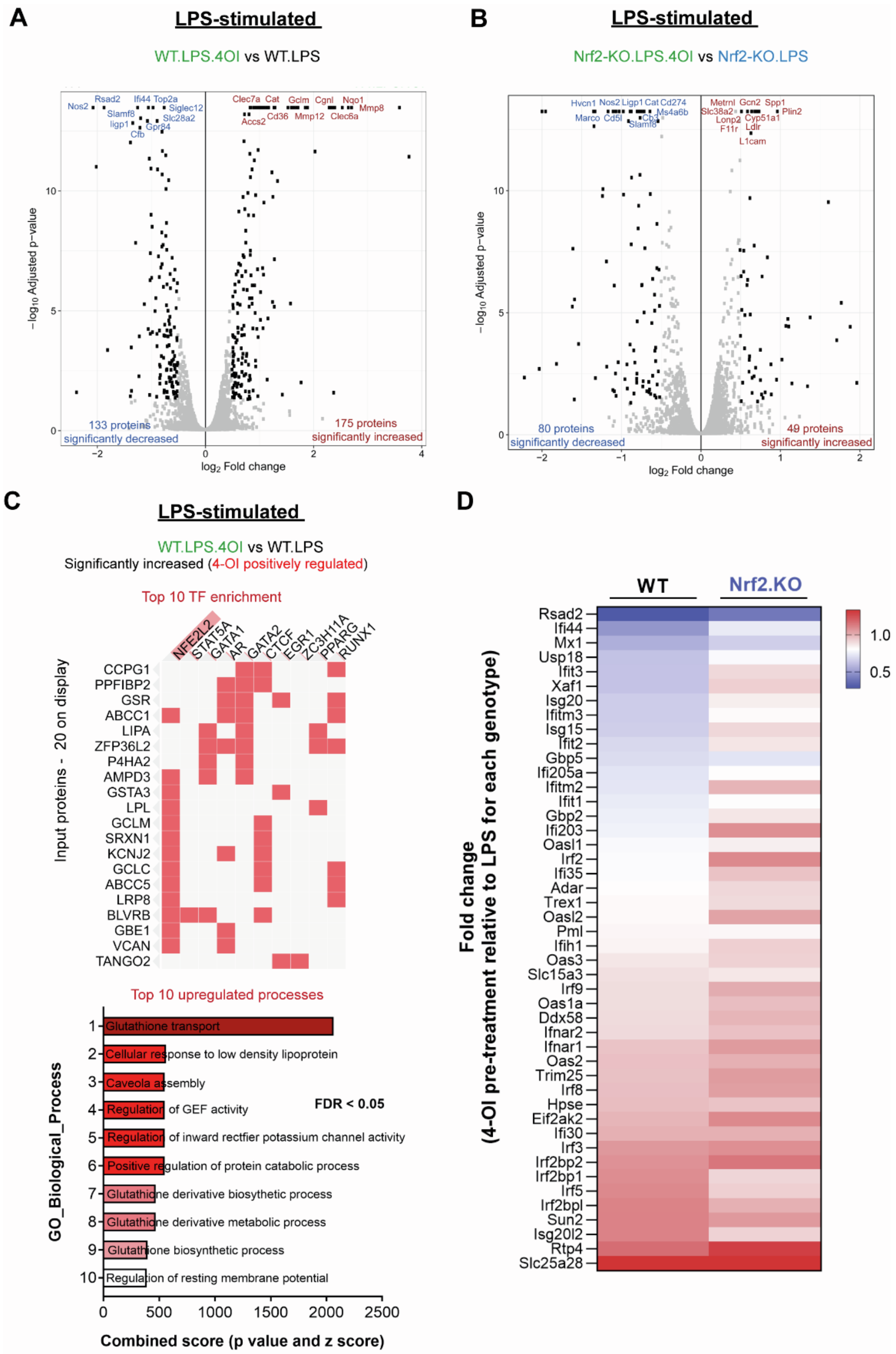

Figure S6 – 4-OI activates Nrf2 and suppresses type I IFN, related to Figure 4

(A) Volcano plot of LPS with 4-OI compared to LPS only (WT)

(B) Volcano plot of LPS with 4-OI compared to LPS only (Nrf2-KO)

(C) Enrichment of TF and GO: biological processes of 4-OI positively regulated targets compared to LPS only (WT). ORA by Enrichr, FDR correction by Bonferroni test. Top processes ranked according to combined enrichment score (p value and Z score).

(D) Heatmap of type I interferon stimulated proteins comparing 4-OI pre-treatment of LPS activated cells to LPS only for each genotype. Data are mean of fold change from 3 biological replicates per condition.

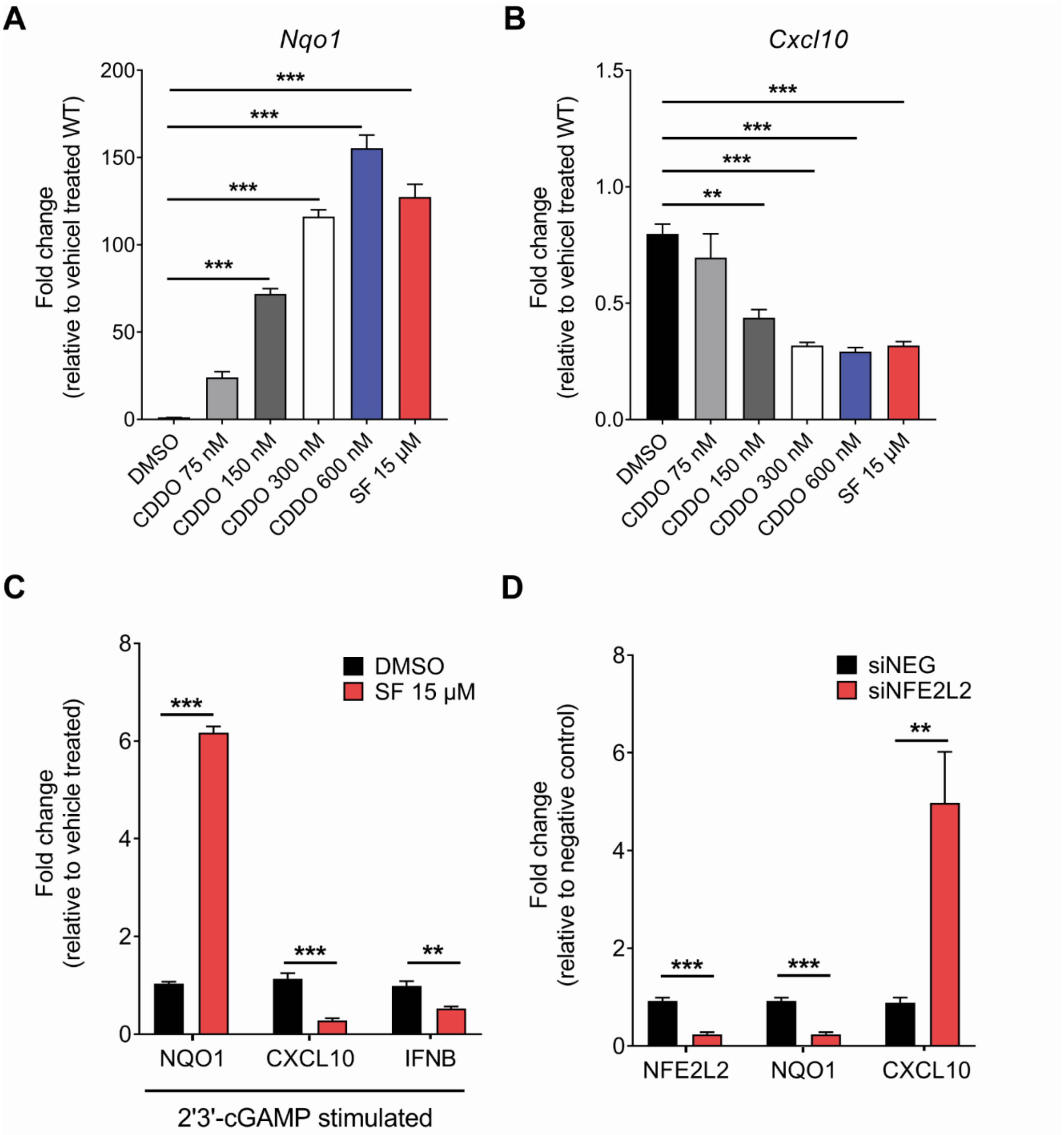

Figure S7 – The pharmacologic Nrf2 activators SF and CDDO downregulate type I IFN response, related to Figure 4

(A) mRNA levels for NQO1 in RAW264.7 cells that had been treated with sulforaphane (SF) or increasing concentrations of CDDO for 24 h (n = 3 biological replicates).

(B) mRNA levels for CXCL10 in RAW264.7 cells that had been treated with sulforaphane (SF) or increasing concentrations of CDDO for 24 h (n = 3 biological replicates). Data are mean ± SEM. p value determined by one-way ANOVA, corrected for multiple comparisons by Tukey statistical test. p <0.05*; p <0.01**; p < 0.001***.

(C) mRNA levels for NQO1, CXCL10 and IFNB in PMA-differentiated THP1 cells that had been treated with SF for 24 h, and then transfected with 2’3’-cGAMP for a further 6 h (n = 3 biological replicates).

(D) mRNA levels for NFE2L2, NQO1 and CXCL10 in PMA-differentiated THP1 cells that had been treated with si*NFE2L2* or siNeg control and harvested 48 h post-siRNA treatment. Note in each case the reciprocal relation between the expression of NQO1 and CXCL10.

Data are mean ± SEM. p value determined by multiple t tests - one per row, corrected for multiple comparisons by Holm-Sidak test. p <0.05*; p <0.01**; p < 0.001***.
